# Yeast Biodiversity in Fermented Doughs and Raw Cereal Matrices and the Study of Technological Traits of Selected Strains Isolated in Spain

**DOI:** 10.3390/microorganisms9010047

**Published:** 2020-12-26

**Authors:** Rosana Chiva, Lorena Celador-Lera, José Antonio Uña, Ana Jiménez-López, María Espinosa-Alcantud, Enrique Mateos-Horganero, Soledad Vega, María Ángeles Santos, Encarna Velázquez, Mercedes Tamame

**Affiliations:** 1 Instituto de Biología Funcional y Genómica (IBFG), CSIC/Universidad de Salamanca, 37007 Salamanca, Spain; rosanachiva@usal.es (R.C.); ajimenez@vlpbio.com (A.J.-L.); maria22_dolores@hotmail.com (M.E.-A.); 2 Departamento de Microbiología y Genética, Universidad de Salamanca, 37007 Salamanca, Spain; lorenacelador@usal.es (L.C.-L.); jaua@usal.es (J.A.U.); solevegafdez7@hotmail.com (S.V.); gemail@usal.es (M.Á.S.); evp@usal.es (E.V.); 3 Repostería Mateos S.A.-Pan del Duero-Mahorpan, 49721 Zamora, Spain; mahorpan@hotmail.com; 4 Unidad Asociada USAL-IRNASA (CSIC), 37008 Salamanca, Spain

**Keywords:** yeasts, cereal matrices, mother doughs of Spain, bakery doughs, RAPD, 5.8S-ITS, D1/D2, phenotypic traits, fungal microbiome

## Abstract

Bakers use pure microorganisms and/or traditional sourdoughs as the leavening agent for making bread. The performance of each starter and the substances produced by the microorganisms greatly affect the dough rheology and features of breads. Modern sourdoughs inoculated with selected lactic acid bacteria and yeasts are microbiologically stable, safer than traditional sourdoughs, and easy to use. However, the commercial repertoire of baker’s yeasts is still limited. Therefore, there is a demand for new strains of yeast species, capable of conferring distinctive traits to breads made from a variety of agri-food matrices, in the design of innovative starters. In this context, we report the first comprehensive study on yeasts isolated from a wide range of fermented doughs, cereal flours, and grains of Spain. Nine yeast species were identified from 433 isolates, which were distributed among separate clades. Moreover, phenotypic traits of potential technological relevance were identified in selected yeast strains. Mother doughs (MDs) showed the greatest yeast biodiversity, whereas commercial *Saccharomyces* starters or related and wild strains often dominated the bakery doughs. A metataxonomic analysis of wheat and tritordeum MDs revealed a greater richness of yeast species and percentage variations related to the consistency, flour type, and fermentation time of MDs.

## 1. Introduction

Yeast-mediated dough fermentation is one of the most ancient and important processes in the bakery industry. These ascomycetous microorganisms are the primary producers of CO_2_ in dough leavening in many bakeries. Baker’s yeast must exhibit a good fermentative metabolism and resist many stresses during this process, but strains produced as industrial starters must also fulfill other requirements [1]. The properties of these domesticated strains, mainly *Saccharomyces cerevisiae*, have significant technological and economic implications [2]. Therefore, new *S. cerevisiae* strains are required for innovative applications in the bakery sector and their study can also lead to an increase in scientific knowledge about the genetics, physiology, and evolution of these microorganisms [3].

Besides pure yeast cultures, sourdoughs are used as starters for dough leavening. A sourdough is a natural leaven consisting of a mixture of flour and water inhabited by indigenous wild yeasts and bacterial species that rise the dough and endows the bread with many of its distinguishing features. Back-slopped sourdoughs (Type 1) are initiated by spontaneous fermentations of a mix of flour and water incubated at ambient temperatures (20 to 30 °C) [4]. After one or a few days, flour and water are added to keep the fermentation active and the refreshment process is repeated several times until the sourdough is considered ready for making bread. This active fermentation (often named chief or mother) is mixed with flour and water to make the final sourdough, which is added to the bread dough during kneading. Additionally, a piece of the chief sourdough (or of the dough after kneading) is reserved for the next bread-making process [3].

Distinct microbial ecosystems are created in Type I sourdoughs. The metabolic diversity of two groups of microorganisms, yeasts and bacteria, enables a wide range of interactions and associations [5,6] that greatly affect the performance of the dough, the nutritional properties, and the overall quality of sourdough breads, including the microbial shelf life [7,8] and functional properties [9,10]. Sourdoughs are widely used to produce “slow breads” (long fermentation time) and “low FODMAP breads” (Fermentable Oligosaccharides, Disaccharides, Monosaccharides, and Polyols), which are increasingly appreciated by consumers due to their nutritional and sensory qualities and effect on human health [10,11,12,13,14,15,16]. However, the relationships among dough ingredients, the conditions of each fermentative process, and the functionality of yeast strains in bread dough have not been studied in depth [17,18]. The fermentative ability and performance of yeasts in each type of dough made of traditional or modern agri-food matrices and their influence on the final quality of breads are objects of intensive study [4,19,20,21,22,23].

Over 30 yeast species have been identified in sourdoughs [24,25,26,27,28]. While intra-dough species diversity is generally low, with only a single dominant yeast species [24], inter-dough diversity is relatively high. The yeast *S. cerevisiae* and species of the neighboring genus *Kazachstania (K. humilis*, *K. exigua)*, or of the more distant genera *Wickerhamomyces anomalus*, *Pichia kudriavzevii*, and *Torulaspora delbrueckii*, are the most geographically widespread species in sourdoughs of Asia, Europe, and in some cases, Africa and Australia. Although *S. cerevisiae* is one of the most commonly described dominant species (~80%), in some sourdoughs it is completely absent or only present at a much lower frequency than other dominant species [3]. Yeast species diversity in sourdoughs has been well described in Italy, Belgium, France, and recently, in Austria [25,26,29,30,31]. In contrast, only a few sourdoughs made in Spain with wheat flour have been prospected for the associated yeast species [32,33,34,35]. Moreover, the genetic diversity of *S. cerevisiae* strains isolated from bakeries, and the phylogeny and diversity of the yeast species in sourdough samples worldwide documented in the scientific literature, were recently reviewed [3] and do not include any samples from Spain.

Similar to what occurs in other fermented foods, the native microbiota of the grains, and consequently, of the flours, plays an important role in the ecosystem of cereal-based foods like sourdough breads [36]. The microbiota of flours may reflect the contamination of the cereal grains, depending on the climatic conditions (temperature and rainfall), insects or fungi contamination, and agricultural practices, among other factors. The flour extraction rate may also have an impact on the yeasts present in flours and sourdoughs [37]. Additionally, brans contain more microbes and metabolites than endosperm (white) flours, which could also be a source of spoilage bacteria and fungi [38].

The study of the yeast species comprising sourdough ecosystems and the phenotypic traits and functionalities of specific strains is relevant for innovation in microbiology, especially with regard to single or mixed starters useful for bread-making. Therefore, the first aim of this study was to isolate in culture yeasts from Type I sourdoughs, known as mother doughs in Spain [35]; leavened doughs from a number of bakeries of different geographical origin; and from raw cereal matrices, flours and grains mostly obtained or cultivated in Spain. Subsequently, identification of the different strains and species was achieved through the analysis of RAPD patterns, which has already been used to analyze the diversity of yeasts isolated from wheat sourdoughs [39], and sequence analysis of the D1/D2 domain and the ITS1-5.8S-ITS2 region of the ribosomal DNA (5.8S-ITS), which has been applied to the identification of yeasts present in sourdoughs in different European countries, particularly in Italy [40,41,42] and France [27,30] and in some mother doughs made in Spain using yogurt and fruits [43].

Different strategies can be applied to analyze the microbiota from complex food matrices. Although culture-dependent techniques are essential to recover yeasts from raw and fermented cereal matrices and study their technological properties, the use of selective media introduces a limiting factor in the identification of viable and non-culturable species in the sourdoughs, which may lead to a biased picture of the yeast communities. For that reason, different culture-independent strategies based on high-throughput sequencing technologies are now widely used in food microbiology. These methods are more sensitive compared with traditional culture-dependent techniques to elucidate the microbiome of fermented food ecosystems and its evolution, and to identify the presence of potential spoilers [43]. Therefore, the second aim of this study was to explore the fungal microbiome of a few MDs using an amplicon-based metagenetic technique [44,45]. This approach consists of the ultra-sequencing of a very small part of the whole-fungal community DNA (5.8S-ITS). This and other high-throughput methods have been applied and the results have been used in the analysis of sourdoughs from four countries [46,47] and in a Global Sourdough Project which includes, to date, only two samples from Spain (http://robdunnlab.com/projects/sourdough/map/ accessed on 24 December 2020).

Recent innovations in bread-making include the use of selected microorganisms in inoculated, modern sourdoughs to improve some of the technological characteristics of breads and elaborate novel products [48,49,50]. Emerging industry and consumer demands have led to the exploitation of alternative raw materials, the use of novel technologies and resources to analyze the microorganisms of the sourdoughs, and an increase in the need to understand in more detail the potential use of non-conventional (*non*-S*accharomyces*) yeast species [49,51]. Consequently, the manufacturers of commercial baker’s yeast and scientists are interested in identifying new yeast species and new *S. cerevisiae* strains to be used in starters for bread [51,52].

The phenotypic diversity of sourdough-associated yeasts and their metabolic properties and functionalities have been explored in many countries [4,25] but, as mentioned above, not yet in Spain. Therefore, the third aim of this study was to explore some phenotypic traits of technological interest in selected strains of different yeast species. Novel yeasts may prove valuable in bread-making processes that employ innovative agri-food matrices, such as flours made from the new cereal tritordeum. This is a hybrid crop obtained by crossing durum wheat (*Triticum durum)* and a wild barley species of Chilean origin (*Hordeum chilense*) [53] that has recently been made available on the market. Tritordeum has lower levels of gluten immunogenic epitopes than wheat [54].

The commercial strains of *S. cerevisiae* have been traditionally selected based on phenotypes suitable for the production of bread, including their fermentative capacity, their ability to generate flavor and aromatic compounds (alcohols, aldehydes, esters, ketones), biomass production, cell growth rate, dehydration, and their tolerance to stress [1,3]. The predominant fermentable sugar in lean doughs is the disaccharide maltose that is produced by the hydrolysis of starch, and the enzymatic maltase activity has been shown to be the rate-limiting factor in maltose fermentation in lean doughs using industrial baker’s yeast [55]. Moreover, it has been shown there is a strong correlation, which is strain-dependent, between the ability of *S. cerevisiae* to ferment sucrose and the capacity to produce and retain glycerol [56]. Glycerol is an important metabolite for osmoregulation under water-stressed conditions [57]. Therefore, the identification of new *S. cerevisiae* strains exhibiting efficient maltose and sucrose metabolism would be of interest to the bakery industry.

Another interesting activity involving *Saccharomyces* and *non-Saccharomyces* species is related to their ability to increase the content of vitamin B in doughs and breads; an increase in the concentration of vitamin B_2_ (riboflavin) was detected in baked products when specific *S. cerevisiae* strains were used as the starter [58]. Additionally, in dough fermentations of rye flour, the use of *K. humilis* (formerly *Candida milleri*) [59] and *T. delbrueckii* lead to increases of 3- to 4-fold in vitamin B_9_ content (folates) [60]. In situ enrichment of vitamin B_9_ has also been reported in oat and barley sourdough fermentations made with *S. cerevisiae* and *K. humilis* [60]. The natural activity of the baker’s yeast *S. cerevisiae* to synthetize vitamin D is currently being exploited by Lallemand Inc. (Montreal, QC, Canada) [61] (https://www.lallemandbaking.com/en/global/brands/instafermvita/ accessed on 24 December 2020).

In addition, sourdough yeasts may have phytase activities that contribute to enhancing the bioavailability of divalent mineral cations (calcium, iron, magnesium, zinc) in baked goods, by degrading the antinutritional phytate–mineral complex particularly abundant in wholemeal flours [62,63]. Additionally, *S. cerevisiae, P. kudriavzevii*, *P. occidentalis*, *K. humilis*, and *K. exigua s*trains isolated from sourdoughs provide phytase activity at optimal dough leavening conditions [64]. Some strains of *W. anomalus* (formerly *Pichia anomala*) and *K. humilis* seems to have, or contribute to, phytase activity [65,66,67,68].

Yeasts may also improve the digestibility of bread, since protease activities have been detected in some sourdoughs when *S. cerevisiae* and *K. humilis* were the dominant species [66]. Accordingly, an increase in the free amino acid pool has been detected in doughs when a strain of *K. humilis* was used as a starter [66]. Moreover, sourdough fermentation technology appears to be successfully applied to obtain gluten-free baked goods [69,70]. Generally, this functionality has been associated with some species of sourdough lactobacilli. However, fermentation of some wheat flour varieties by a strain consortium of *Enterococcus mundtii* QAUSD01 and *W. anomalus* QAUWA03 acts synergistically in reducing the gliadin content of dough [71].

The use of sourdough yeasts greatly contributes to the enhancement and complexity of flavors, and the use of some strains of *T. delbrueckii* and *Saccharomyces bayanus* produce a distinctive aromatic profile in breads [72]. In addition, a systematic study using several combined consortia among a set of seven *non-Saccharomyces* yeasts [73] revealed that *Kazachstania gamospora* and *Wickerhamomyces subpelliculosus* produce a novel aromatic flavor profile in fermented doughs and breads, as well as an increase in stress-tolerance to sugar and salt [52]. An increase in the content of esters, aldehydes, and other aromatic compounds was detected in doughs and breads made with a mixed starter of *Meyerozyma guilliermondii, P. kudriavzevii*, and *Lactobacillus*-specific strains [74]. Moreover, the presence of *W. anomalus* and *M. guilliermondii* seems to be related to the extended shelf life of sourdough breads [75,76].

Our aims were to explore the yeast community in fermented and raw cereal matrices in Spain, characterize yeasts isolated in cultures at the species and strain level, investigate phenotypic traits of interest in selected strains, and analyze the fungal microbiome in some mother doughs. In this work, we have explored the wealth of yeast species naturally present in the exophytic cover of new and traditional cereal grains from a number of geographical or ecological origins, in white and wholemeal flours (wheat, tritordeum, rye, spelt), and in 16 wheat doughs from bakeries located mainly in the north-western Spanish region of Castilla y León. We have also analyzed a variety of seven firm and seven liquid Type I mother doughs (MDs) made by the same baker, as well as seven additional MDs obtained elsewhere. Herein, we report the genetic identification of the yeast species isolated from the wide range of cereal matrices indicated above, the classification of the isolates in clades, the properties and potential technological relevance of a number of selected strains, and the evolution of the fungal microbiome in a few selected mother doughs.

## 2. Materials and Methods

### 2.1. Fermented and Raw Matrices Prospected for Yeast Isolation

#### 2.1.1. Type I Mother Doughs

Fourteen mother doughs (MDs) obtained by spontaneous fermentations of cereal flours were propagated for about a month with daily back slopping (Repostería Mateos S.A., Mahorpan—Pan del Duero—Entrala, Zamora, Spain). The 14 traditional MDs (Table 1) were propagated from an initial firm dough with a dough yield (DY) of 150–160 (DY = [flour mass + water mass] × 100/flour mass).

Seven “firm” MDs with DY ~150–160 (odd numbers, MD1 to MD13; Table 1) were propagated using the following back slopping procedure: (i) 30 g of each flour was mixed with 18 g of tap water and 2% table salt and the dough was allowed to ferment for 5 to 6 days at 20 °C; (ii) at this time point, 20 g of the same flour, 10 g of water, and 2% salt were added to the previous dough and allowed to ferment for 24 h at 20 °C. Then, at this fermentation point (6–7 days, ~1 week), 10 g of the fermented MD was removed and used for the isolation of yeast species on solid cultures, which was considered as back slopping step 1 (BS1). This is also called the pre-mother dough phase, which usually takes about 1 week to develop full fermentative capacity. (iii) The remaining dough was mixed thoroughly with 20 g of flour, 10 g of water, and 2% salt and incubated for 24 h at 20 °C. The daily back slopping procedure (6–24 h of fermentation time) was repeated for ~30 days (~1 month) and was considered as the Final Procedure sample (FP), from which 10 g of MD was removed to isolate the yeast strains.

Seven “liquid” MDs with DY ~280 (even numbers, MD2–MD14; Table 1) were started and propagated as described above. However, in this case, only 10 g of flour was used, to which 10 g of flour and 18 g tap water were added in each step of back slopping. Samples were removed at steps BS1 and FP for yeast strain isolation.

Seven additional MDs (MD15–MD21; Table 1) were collected from artisan bakeries, as well as from private bakers that generally start their bread-making process using a piece of a final MD from a previous process, refresh it through back slopping, and who may have used baker’s yeast.

The 21 MDs prospected for yeast species and the corresponding flours are listed in Table 1.

#### 2.1.2. Leavened Doughs from Bakeries

Leavened bakery doughs (BDs) are generally started using a strain of a commercial baker’s yeast (Type 2) or with a commercial yeast combined, or not, with a portion of a previous MD followed by back slopping (Type 3) [4].

The 16 bakeries included in this study are located in towns or villages in five provinces belonging to the autonomous region of Castilla y León, located in north-western Spain (Table 2). Most of the BDs were made with soft, bread-quality wheat flours (W90-200) in one of the two ways indicated above, and bakers very often store samples of a previous dough at room temperature or in the refrigerator until next use. In each bakery, fresh pieces of ~10 g of a final leavened dough were withdrawn and two samples (from the surface and inside) were taken twice in the bread-making rooms. Samples were collected and stored at 4 °C upon arrival until culture-based analysis was performed and those from Zamora were also brought to the laboratory in refrigerated conditions (Asezpan, Association of Bread Manufacturers, Zamora, Spain)

#### 2.1.3. Cereal Grains and Flours

Ten types of grains from four cereal crops (rye, barley, wheat, and oat) were provided by the milling factory Emilio Esteban S.A. (Valladolid, Spain) and grains from tritordeum crops, harvested from several geographic locations, were provided by Agrasys S.L. (Barcelona, Spain). Twenty types of flours were obtained from several companies (Table 3).

### 2.2. Yeast Isolation, Diversity Analysis, Species Identification, and S. cerevisiae Strain Distinction

#### 2.2.1. Yeast Isolation

Samples of fermented and raw cereal matrices were prospected for wild, indigenous yeasts that could be isolated by conventional culture-dependent techniques [77].

To this end, samples of 10 g of each MD or fermented baking dough (Table 1 and Table 2) and 2 g of intact (not milled) cereal grains or selected flours (Table 3) were homogenized in 90 mL of sterile peptone water (1 g/L peptone, 8.5 g/L NaCl) in 250 mL flasks and incubated at 28 °C for 1 h with shaking (200 rpm). For yeast isolation, samples were collected by centrifugation at 10,000 rpm and ten-fold dilutions of the supernatants were spread as 0.1 mL aliquots on SDCA plates (Sabouraud Dextrose Chloramphenicol agar plates, Merck Life Science S.L.U., Sigma Co., Madrid, Spain) that were incubated at 28 °C for ~4 days [26]. At this time, the colonies were counted and transferred to new plates for yeast isolation and identification using genotyping techniques. Approximately 10 colonies were randomly picked from plates and cells from each colony were observed under a phase contrast microscope to select those with a morphology most likely to correspond to yeasts.

#### 2.2.2. DNA Extraction

DNA was extracted from yeast cells grown on YPD plates (0.5% yeast extract, 1% glucose, and 2% agar) for 48 h at 28 °C. Cells were suspended in sterile water and collected by centrifugation in a Micro-Spin centrifuge at 5000× *g* at room temperature and then washed with 200 μL of 0.1% aqueous solution of sarkosyl [78]. Total DNA was extracted with 100 μL of 0.05 M NaOH (DNA-free) and heat at 100 °C for 4 min. The samples were placed in an ice bath, and 900 μL of water was added to each microtube and mixed thoroughly. After an additional centrifugation at 4000× *g* for 3 min, 700 μL of the supernatants were harvested and frozen at –20 °C until used. The quality and concentration of the extracted DNA was checked by Nanodrop ND-1000 (Fisher Scientific, Waltham, MA, USA).

#### 2.2.3. RAPD Pattern Analysis

RAPD fingerprinting patterns were obtained by PCR using total yeast DNA as the template, the M13 primer (5′-GAGGGTGGCGGTTCT–3′), and Dream Taq-polymerase (Thermo Fisher, Madrid, Spain) [79]. PCR conditions were as follows: preheating at 95 °C for 1 min 30 s; 35 cycles of denaturing at 95 °C for 30 s; annealing at 36 °C for 1 min and extension at 75 °C for 2 min; and two final extension steps at 75 °C for 7 min and 60 °C for 10 min. The PCR products were conserved at 5 °C and 10 µL of each sample were electrophoresed on 1.5% (*w*/*v*) agarose gel in TBE buffer (100 mM Tris, 83 mM boric acid, 1 mM EDTA, pH 8.5) at 6 V/cm, stained in a solution containing 0.5 μg/mL ethidium bromide and photographed using a Gel Doc XR (Bio-Rad, Madrid, Spain). Standard (GeneRuler 1Kbp Plus DNA Ladder, Thermo Fisher, Madrid, Spain) was used as a size marker. A dendrogram was constructed based on the matrix generated using the UPGMA (Unweighted Pair Group Method with Arithmetic mean) hierarchical clustering method and Jaccard’s coefficient with Bionumerics version 4.0 software (Applied Mathematics, Austin, TX, USA).

#### 2.2.4. Genus and Species Identification

The D1/D2 domain of the 28S rDNA gene was amplified and sequenced using D1 (5′-AGTAACGGCGAGTGAA(GC)CG-3′) and D2 (5′-CCMAAACYGAYGCTGGCCC-3′) primers and the 5.8S-ITS internal transcribed region of the rDNA with ITS1F (5′-TCCGTAGGTGAACCTGCGG-3′) and ITS4R (5′-TCCTCCGCTTATTGATATGC-3′) primers [80]. Amplification was performed with an AmpliTaq reagent kit (Perkin Elmer Biosystems, Foster City, CA, USA) following the manufacturer’s instructions. PCR amplification was carried out as follows: pre-heating at 95 °C for 9 min; 35 cycles of denaturing at 95 °C for 1 min; annealing at 55 °C for 1 min and extension at 72 °C for 1 min; and a final extension at 72 °C for 7 min. The PCR product was electrophoresed at 6 V/cm on 1% agarose gels with TBE buffer and the gels were stained in a solution containing 0.5 μg/mL ethidium bromide. The DNA bands were purified directly from the gel by room temperature centrifugation in Eppendorf tubes with a special filter (Millipore Co., Burlington, MA, USA) for 10 min at 5000× *g* according to the manufacturer’s instructions. When necessary, the 5.8S-ITS amplicons were cloned before sequencing using the Zero Blunt TOPO PCR Cloning Kit (Thermo Fisher, Madrid, Spain) following the manufacturer’s instructions. The sequence reactions were performed on an ABI377 sequencer (Applied Biosystems Inc., Foster City, CA, USA) using a BigDye terminator v3.0 cycle sequencing kit as supplied by the manufacturer. The sequences obtained were compared with those from the GenBank using the BLAST program [81] and are currently available in the GenBank under accession numbers MT645338–MT645397 (D1/D2 region) and MT645398–MT645457 (5.8S-ITS region).

Pure yeast cultures were grown under sterile conditions in YPD at 28 °C for 24–48 h and stored at −80 °C in laboratory ultra-freezers in sterile capped plastic tubes in 20% glycerol until further use. All the newly isolated yeasts were included in the PANLEV Collection of Microorganisms of the IBFG (CSIC—University of Salamanca, Salamanca Spain) and Microbiology and Genetics Department (University of Salamanca, Salamanca, Spain).

#### 2.2.5. Distinguishing *S. cerevisiae* Strains

Delta-LTR sequences of Ty transposons (δ) are targets for the identification of polymorphisms, and the inter-delta method is often routinely used for distinguishing different yeast strains. PCR amplifications of the inter δ regions were performed in a Biometra Thermocycler T-Gradient Thermo Block (Analytik Jena AG, Jena, Germany) using genomic DNA of the *S. cerevisiae* strains as the template and δ12 (5′-TCAACAATGGAATCCCAAC-3’) and δ21 (5′-CATCTTAACACCGTATATGA-3′) primers, all as described in Legras and Karst [82]. PCR reactions were carried out in 50 μL reaction volumes containing 1 unit of Ecotaq polymerase (Ecogen, Madrid, Spain), 5 μL Ecotaq polymerase 10× buffer, 2.5 μL of 50 mM of MgCl_2_, 1 μL of 0.1 mM of each dNTP (Merck Life Science S.L.U, Sigma Co., Madrid, Spain), and 5 μL of 10 μM of primers δ12 and δ21. Amplification was performed for 35 cycles under the following conditions: after 10 min of initial DNA denaturation at 95 °C, each cycle consisted of 30 s denaturation at 95 °C, 30 s primer annealing at 46 °C and 1.30 min primer extension at 72 °C, followed by a 10 min final extension step at 72 °C. The PCR products were electrophoresed on a 2% agarose gel (*w*/*v*) in 1X TBE buffer, detected after ethidium bromide staining (10 μg/mL) using a Gel Doc 2000 apparatus (Bio-Rad, Madrid, Spain) and the footprint patterns were compared.

Minisatellite-like fingerprints were obtained according to Siesto et al. [83]. Some *S. cerevisiae* strains are characterized for their allelic variation at four minisatellite loci that cause length polymorphisms and characteristic finger printings [84,85,86]. Genomic DNAs were analyzed using PCR to identify minisatellite-like sequences in the *AGA1* and *SED1* genes. PCRs were carried out in 25 μL reaction volumes containing 0.5 units of Ecotaq polymerase (Ecogen, Madrid, Spain), 2.5 μL Ecotaq polymerase 10× buffer, 1.25 μL of 50 mM of MgCl_2_, 0.5 μL of 0.1 mM of each dNTP (Merck Life Science S.L.U, Sigma Co., Madrid, Spain), and 1.25 μL of each primer at 10 μM. Amplification was performed for 35 cycles under the following conditions: after 10 min of initial denaturation at 95 °C, each cycle consisted of 1 min denaturation at 95 °C, 1 min primer annealing at 64 °C and 1 min primer extension at 72 °C, followed by a 10 min final extension step at 72 °C. The PCR products were analyzed by electrophoresis on a 1% agarose gel in 0.5X TBE buffer. Samples of the PCR products were digested with restriction enzymes *Hpa*II *(SED1*) and *Alu*I (*AGA1*) [86] electrophoresed on 2% agarose gels, and the patterns of the DNA fragments were compared and photographed as previously indicated.

### 2.3. Metataxonomic Analysis of Selected MDs

A metataxonomic analysis was performed for four selected MDs to explore their respective fungal microbiomes, which consists of the taxonomic analysis of metagenetic sequences. This method involves PCR-based amplification of a specific genomic region using whole-community DNA, followed by the high-throughput sequencing of the amplicons obtained (Biome Makers, Valladolid, Spain). DNA was extracted from 1 g of each of the four MD samples using the DNeasy PowerLyzer PowerSoil Kit (Qiagen, Hilden, Germany). DNA libraries were prepared by amplifying the rDNA 5.8 ITS1 sequences using Biome Makers^®^ custom primers (Patent WO2017096385). An average of 150,000 reads was generated per sample using 2 × 301 bp paired-end sequencing with an Illumina MiSeq platform (Illumina, San Diego, CA, USA). In order to validate the procedure, a sterilized Milli-Q water sample (instead of gDNA) was included as a control in the DNA extractions and downstream PCR amplification procedures. Raw sequences were analyzed using V-search default parameters. Briefly, raw paired-end fast sequences were merged, filtered by expected errors at 0.25, dereplicated, and sorted by size. The chimera sequences were filtered out and the remaining sequences clustered into 97% identity Operational Taxonomic Units (OTUs). Only the groups with at least two sequences were considered for further analysis. Combined sequences were then mapped to the list of OTUs with at least 97% similarity, resulting in a table with OTU sequences quantified per biological sample. OTUs were classified with a curated UNITE version.

### 2.4. Phenotypic Analysis

#### 2.4.1. Yeast Strains and Culture Conditions

A number of yeast isolates, representative of each species belonging to our PANLEV culture collection, were analyzed for several phenotypic traits. The yeast strains submitted to the phenotypic assays belong to *S. cerevisiae* or *non-Saccharomyces* species previously identified by PCR–RFLP and sequence analysis of the rDNA region (Section 2.2.4).

Yeast cells were grown on rich YPD medium plates (yeast extract 2%, peptone 2%, glucose 2%, bacto-agar 2%). The culture conditions in the liquid YPD media and growth determinations were as described in Codón et al. [87]. Briefly, yeasts were inoculated into 20 mL tubes containing 5 mL of liquid YPD and incubated at 28 °C with rotatory shaking of 250 rpm until the stationary phase was reached (about 10^8^ cells/mL). Flasks of 1 L with 200 mL of medium were prepared and inoculated with the stationary-phase cultures until reaching an initial optical density of 0.1 at 600 nm (OD_600_). After cell inoculation, flasks were incubated at 28 °C with shaking. Growth was determined by measuring the increase in turbidity (OD_600_) using a Hitachi U2000 (Hitachi High-Tech Analytical Science, Uedem, Germany). Representative strains of commercial baker’s yeasts were used as the reference in the phenotypic tests.

#### 2.4.2. Leavening Ability of Yeasts in Doughs

The leavening ability of yeast strains under laboratory conditions was evaluated according to Rincón et al. [88]. Cells were grown in YPD at 28 °C for 12–24 h at 200 rpm in a shake up to a cellular density of about 10^8^ cells/mL. Doughs were prepared in 20 mL graduated glass tubes containing 7 mL of distilled sterile water [33]. Four grams of wheat flours (specific deformation work (alveograph value) W150 or W200 × 10^3^ ergs for plain doughs) or tritordeum W87–W110 flours were mixed thoroughly and inoculated with 100 mg of fresh cells of the yeasts previously grown in YPD [wet weight 1.5 × 10^7^ cells/mg per gram of flour]. The graduated tubes were incubated without shaking at 28 °C. Dough leavening was monitored every 10–15 min for ~90 min and estimated, in mL, as the increase that the dough cylinder (V = π × r^2^ × h) had reached at each time point.

#### 2.4.3. ANKOM-Gas Production

The gas produced during the fermentation process was analyzed using an ANKOM RF Gas Production Measurement System (Ankom technology, Fairport, NY, USA), which allows the measurement of pressure and temperature inside bottles connected to a wireless module. These CO_2_ production measurement tests were carried out with yeasts grown in 50 mL of YPD at 28 °C for 48 h in an orbital shaker at 200 rpm. The OD_600_ of the cultures was measured and a number of cells equivalent to a dry weight mass of ~30 mg/mL was collected (OD_660_ ~0.35 mg of cells [dry weight]/mL) [89]. Fifteen milliliters of the yeast mixture was poured into a 250 mL bottle and placed in a water bath at 30 °C. After 15 min, 15 mL of model liquid dough (MLD) prewarmed at 30 °C was added. The pressure increase inside the bottle was automatically measured over 18 h every 15 min in the ANKOM system. The MLD solution was prepared as follows, according to Panadero et al. [89], with a formula provided by Lesaffre International Group (Marcq-en-Baroeul, France). First, a 5X concentrated nutrient solution containing 5 g of MgSO_4_ 7H_2_O, 2 g of KCl, 11.75 g of (NH_4_)_2_HPO_4_, 4 mg of thiamine, 4 mg of pyridoxine, and 40 mg of nicotinic acid in a final volume of 250 mL of 0.75 M citrate buffer (pH 5.5) was prepared. Twenty milliliters of the concentrated nutrient solution were added to a tube containing 0.5 g of yeast extract, 3 g of glucose, 9 g of maltose, and 12 g of sorbitol. Distilled water was added to a final volume of 100 mL, and the solution was filter sterilized. Finally, the amount of pressure produced was expressed in units of gas volume (CO_2_) applying the gas formula: mL gas produced = P (pressure in KPa) × V (volume in L) × R (gas constant) × T (temperature, K) × 22.4 L/mol × 1000.

#### 2.4.4. Hydrolytic Enzyme Assays

Maltase activity (alpha 1-4 α-glucosidase hydrolyzes 1,4-linked α-D-glucose residues with the release of α-D-glucose) was quantified in the whole cell extracts (WCE) of *S. cerevisiae* strains using p-nitrophenyl-a-D1,4-glucopyranoside (pNPG) as a substrate. To prepare the WCE, cells were grown to the early exponential phase (OD_600_ of 0.8–1) under repressing or derepressing conditions in YPD or YPM (2% yeast extract, 2% Bacto peptone) and 2% glucose or 2% maltose, respectively. Samples of 5 mL were harvested from cultures and the cells were suspended in 300 μL of breaking buffer (0.1 M Tris-HCl pH 8.0, 20% (*v*/*v*) glycerol, 1 mM 2mercaptoethanol (Merck Life Science S.L.U, Sigma Co., Madrid, Spain), and 2 mM Phenyl-Methyl-Sulphonyl-Fluoride (PMSF, Merck Life Science S.L.U., Sigma Co., Madrid, Spain) in 90% ethanol) and transferred to tubes containing 1g of glass beads. The mixture was vortexed for two periods of 2 min at 4 °C, centrifuged at 12,000 *g* at 4 °C for 15 min and the supernatant was used for further analysis. Protein amounts in the WCE were determined by the Bradford protein assay [90], using a commercial dye reagent and bovine serum albumin as a protein standard. To quantify maltose activity, a reaction mixture containing 50 μL of the supernatant (0.5–1 mg/mL protein) was added to 400 μL of buffer Z (0.06 M Na_2_HPO_4_·7H_2_O, 0.04 M Na_2_HPO_4_·H_2_O, 0.01 M KCl, 0.001 M MgSO_4_·7H_2_O, 0.05 M 2-mercaptoethanol) with 100 μL of substrate PNPG (4 μg/μL) incubated at 30 °C. Once the mixtures had turned yellow, the reaction was stopped by adding 250 μL 1 M Na_2_CO_3_ and the time that had elapsed since the start of the reaction was recorded. The absorbances of the samples were spectrophotometrically determined at 420 nm (OD_420_), taking into consideration the dilution factor. Units of maltase activity were calculated and expressed as nanomoles of para-nitrophenol liberated from the pNPG per minute x mg protein.

Invertase activity (the enzyme that catalyzes the hydrolysis of sucrose, producing a mixture of fructose and glucose, which is called inverted sugar) was estimated in yeast WCE using a colorimetric method involving the 3,5-dinitrosalicylic acid (DNS) assay [91]. To prepare the WCE, cells were grown to the early exponential phase (OD_600_ of 0.8–1) under repressing or derepressing conditions in YPD or YEPS (2% yeast extract, 2% Bacto peptone) and 2% glucose or sucrose, respectively. The reaction mixture containing 0.1 mL of the whole cell extract (0.5–1 mg/mL protein), 1 mL sucrose 0.2 M, 2 mL acetate buffer (0.1 M, pH 5.0), and 1 mL of water was incubated at 55 °C for 30 min. Then, 0.1 mL of the reaction mixture was transferred to a new tube and 1 mL of DNS reactive (DNS 0.1% *w*/*v*, sodium potassium tartrate 30% *w*/*v*, NaOH 0.4 M) was added; the reaction tubes were then boiled for 10 min and cooled in an ice bath. The absorbance of the samples was determined spectrophotometrically at 570 nm (OD_570_), taking into consideration the dilution factor and the concentration of reducing sugars estimated using a glucose standard curve as reference. Units of invertase activity were calculated and expressed as millimoles of glucose liberated per min x mg of WCE protein.

#### 2.4.5. Assays for Vitamin Requirement and Riboflavin Production

The vitamin requirements of the yeast strains were determined using three defined minimum media based on a synthetic medium described in Wickerham et al. [92]. The vitamin-free minimum medium for yeast, VFMMY, contains glucose, mineral salts, trace elements, and the precursor of the membrane phospholipid inositol. The VFMMY (+) was supplemented with one or more vitamins to determine the specific requirements of each of the analyzed strains in relation to the MMY, which contains all the B vitamins: thiamine (B_1_), riboflavin (B_2_), nicotinic acid (B_3_), calcium pantothenate (B_5_), pyridoxine (B_6_), biotin (B_7_), folic acid (B_9_), and p-aminobenzoic acid (B_10_). The media were dispensed in 96-well microplates (200 μL per well) and inoculated in duplicate with 5 μL of cells from a 10-fold diluted pre-culture of each yeast strain grown in MMY for 4 days (OD600~1.0). The microplates were incubated at 28 °C and cell growth was followed by measuring the OD_600_ of the cultures every day for 5 days using a Multiskan GO microplate spectrophotometer (Thermo Fisher, Madrid, Spain). The yeast strains with vitamin requirements grew (OD_600_ 1.1 ± 0.3) in the respective MMY and the VFMMY+ supplemented media containing the required vitamin/s, but not in the non-supplemented VFMMY. 

The riboflavin secreted by producer yeasts strains was visually estimated in 2.5 mL of the MMY culture medium (filtered using 0.22 μm pore filters) under an ultraviolet light lamp (360 nm wavelength) after 4 days of incubation at 28 °C. The identity of the vitamin was confirmed by thin-layer chromatography (data not shown) on silica gel plates (Merck Life Science S.L.U, Sigma Co., Madrid, Spain) using the mobile phase *n*-butanol: acetic acid: water (10:3:7 *v*/*v*) [93] and synthetic pure riboflavin as the control (Merck Life Science S.L.U, Sigma Co., Madrid, Spain). The amount of riboflavin produced by each yeast strain was spectrophotometrically quantified at OD_472_ in microtiter plates (Nunc™, Thermo Fisher, Madrid, Spain). A calibration curve was made with serial dilutions of a synthetic riboflavin stock aqueous solution (0.1 mg/mL *w*/*v*). Samples of 0.2 mL from yeast cultures grown in microtiter plates were filtered and the quantity of riboflavin produced by each yeast strain was spectrophotometrically determined by interpolation of the OD_472_ values in the calibration curve. To evaluate the influence of iron on riboflavin production, increasing amounts of F were added to the MMY [93].

#### 2.4.6. Assays for Extracellular Enzyme Activities

The enzymatic activities of esterase, protease, glyadinase, peptinase, amylase, cellulase, and β-glucosidase were assayed on plates of solid yeast nitrogen base without amino acids medium (YNB-AA) (Formedium™ Ibian Technologies, Zaragoza, Spain). This medium was supplemented with the appropriate substrate for each enzyme according to the procedure already described by Carrasco et al. [94]. The β-glucosidase activity was evaluated as described in Strauss et al. [95]. Yeasts strains were grown on YPD plates at 28 °C for 48 h and then replica plated to YNB-AA containing each specific substrate (Merck Life Science S.L.U., Sigma Co., Madrid, Spain).

Phytase and cellobiase activities were estimated in cultures grown in microtiter plates, using phytic acid and cellobiose as the sole phosphorous and carbon source, respectively. The phytase test was performed as described by Oltospore et al. [96] with some modifications. Yeasts were inoculated in 2 mL of YPD medium and cells from ~1 mL of cultures (OD_600_~2.0) were harvested by centrifugation, washed two times with 1 mL of ultrapure water, resuspended in 2 mL YNB-AA and YNB-AA without KH_2_PO_4_ (YNB-AA-P) (Formedium^TM^ Ibian Technologies, Zaragoza, Spain), and the final suspension was incubated at 28 °C for 24 h in order to deplete the intracellular phosphate content. Then, the cell suspensions were diluted to an OD_600_ ~0.01 and 10 μL from the dilution solution was inoculated in triplicate into 200 μL of media YNB-AA-P, YNB-AA, and YNB-AA-P supplemented with 0.2% (*w*/*v*) phytic acid dipotassium salt (YNB-AA-P+Pa) in the wells of microtiter plates (Nunc™, Thermo Fisher, Madrid, Spain). Two percent glucose was used as the carbon source in the three media. Growth was followed every day by measurement of the increase in turbidity in the laboratory medium (OD_600_). Phytase activity was considered positive when yeast strains grew efficiently in YNB-AA-P+Pa, as in YNB-AA (OD_600_~1.2 ± 0.2) and did not grow in the YNB-AA-P without phosphorous. A similar procedure was used to detect cellobiase activity, but in this case, the media used were YNB-AA and YNB-AA supplemented with 2% cellobiose disaccharide (YNB-AA+C). Additionally, the cells that were to be inoculated in YNB-AA+C were previously grown in YNB-AA for 24 h in order to deplete the intracellular glucose content.

## 3. Results

### 3.1. Genotyping of the Yeast Isolates

In this work, 433 presumptive wild yeasts were isolated and analyzed from 21 mother doughs (MDs, Table 1), 16 bakery leavened baking-doughs of the Castilla y León region of Spain (BDs, Table 2), 10 types of cereal grains, and 20 flours (Table 3). The yeast isolation procedure is described in the Materials and Methods (Section 2.1.1). The yeast isolates are summarized in Table S1 according to the laboratory codes, the species, and the source.

#### 3.1.1. Analysis of the 5.8S-ITS and RAPD Patterns

The amplification of the 5.8S-ITS rDNA region of the yeasts analyzed yielded six types (I to VI) of banding patterns of different sizes (Supplementary Materials, Figure S1). The RAPD patterns of the strains displaying each one of these 5.8S-ITS types were analyzed separately, and the resulting dendrograms are presented in Figures S2–S7. After considering a similarity cut-off of 80% in all cases, we obtained 60 RAPD groups encompassing the strains with different 5.8S-ITS fragment sizes (Table S2). Specifically, 13, 4, 13, 7, 18, and 5 RAPD groups were established for 5.8S-ITS types I, II, III, IV, V, and VI, respectively. A representative strain from each RAPD group was selected for sequencing the 5.8S-ITS and D1/D2 regions.

#### 3.1.2. Analysis of the 5.8S-ITS and D1/D2 Regions

The results of these analyses showed that yeast isolates displaying different 5.8S-ITS types belonged to different genera, as shown in Table 4 and Supplementary Figures S2 (Type I; *Kazachstania*), S3 (Type II; *Meyerozyma*), S4 (Type III; *Pichia*), S5 (Type IV; *Saccharomyces*), S6 (Type V; *Torulaspora*), and S7 (Type S6; *Wickerhamomyces*).

(I) The yeast isolates displaying a 5.8S-ITS type I pattern belonged to several species of the genus *Kazachstania*. Those from RAPD groups A to G and I to K belonged to the species *K. bulderi* with 100% similarity in both regions with respect to its type strain. Those from RAPD group H showed 97.7% and 100% similarity, respectively, with respect to the type strain of *K. humilis*. The isolates from groups L and M presented near to 100% similarity in the two regions analyzed with respect to the type strain of *K. servazzii*, although they presented slight differences in the 5.8S-ITS sequences.

(II) The yeast isolates displaying 5.8S-ITS type II belonged to two species of the *Meyerozyma* genus. Those from RAPD groups A and C presented similarity values equal to or near to 100% in both regions analyzed with respect to the type strain of *M. guilliermondii* and those from groups B and D with respect to the *M. carpophila* type strain, although they have slight differences in their 5.8S-ITS sequences.

(III) The yeast isolates displaying 5.8S-ITS type III belonged to the species *Pichia fermentans*. All isolates showed similarity values near to 100% in the D1/D2 region and equal to or near to 100% in the 5.8S-ITS region, except in the case of those from RAPD group A, H, and J that presented 98.4% similarity values.

(IV and V) The yeast isolates displaying 5.8S-ITS types IV and V belonged to *S. cerevisiae* and *T. delbrueckii*, respectively. All of these isolates presented similarity values equal to or near to 100% in the two regions analyzed with respect to the species’ type strains.

(VI) Finally, the yeast isolates displaying 5.8S-ITS type VI belonged to the species *W. anomalus*. Most of the isolates presented similarity values equal or near to 100% in the two regions analyzed with respect to the type strain of this species, except in the case of those from RAPD group B for the 5.8S-ITS region with a similarity value of 98.7%.

### 3.2. Distribution of the Yeast Species and Isolates

**Figure 1:**
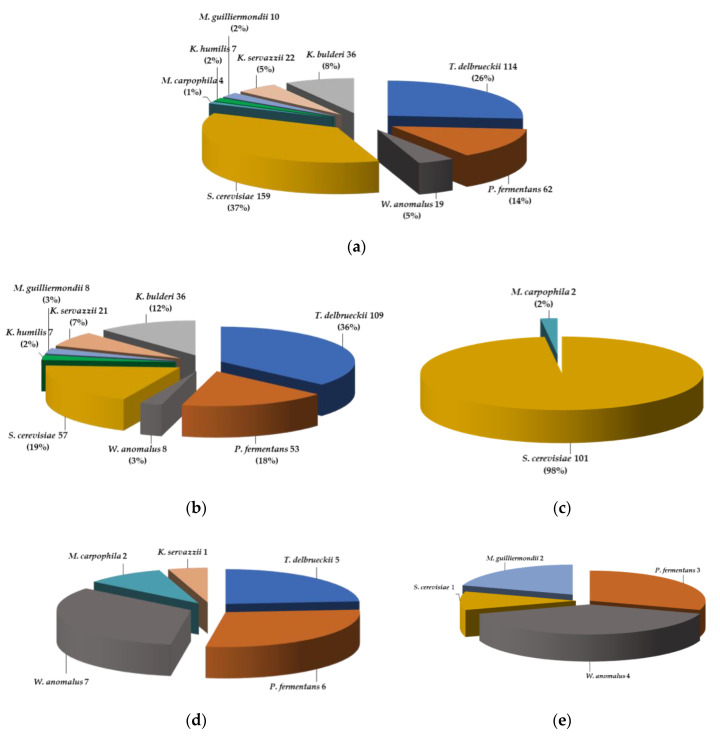
Species distribution among the 433 yeast isolates recovered on culture plates. (**a**) 67 cereal matrices (Table S1); (**b**) 21 Mother Doughs (Table 1); (**c**) 16 Bakery Doughs (Table 2); (**d**) 20 flours (Table 3); and (**e**) 10 types of cereal grains (Table 3).

A systematic study on the yeasts isolated from mother doughs, bakery doughs, and a wide range of raw cereal matrices from Spain is reported here for the first time. The representativeness of the yeast species (as the frequency of microbiological isolation) in the collection of isolates from the matrices that were analyzed (Table S1) is represented in the pie charts of Figure 1.

The 433 isolates belong to nine yeast species: *S. cerevisiae* (159), *T. delbrueckii* (114), *P. fermentans* (62), *K. bulderi* (36), *K. servazzii* (22), *W. anomalus* (19), *M. guilliermondii* (10), *K. humilis* (7), and *M. carpophila* (4) (Figure 1a). The number of yeasts isolated from all matrices is represented in Figure 1a and by each type of matrix in Figure 1b–e and the respective species are represented.

The diversity of yeast species in the isolates recovered from each matrix was as follows:

Isolates from mother doughs (MDs, Table 1) belong to one or more yeast species (Table S1) that may or not represent the most abundant or dominant species in each MD (Table S3a,b). Eight yeast species were isolated from a number of MDs: *S. cerevisiae* from 14 MDs (1–5, 9, 11, 13–15, 17, 18, 20, 21); *P. fermentans* from 14 MDs (1–8 and 10–14, 17); *T delbrueckii* from 12 MDs (1–5, 7, 9–14); *K. servazzii* from 9 MDs (2, 3, 5–9, 11, and 13); *K. bulderi* from 7 MDs (2, 4, 6, 8, 10, 12, and 14); *W. anomalus* from 4 MDs (1, 5, 7, and 16); *K. humilis* from 2 MDs (18 and 19)*;* and *M. guilliermondii* from MD6.

Some correlations were observed among the frequency of species isolation and the consistency or the age (back slopping steps of fermentation, BS1 ~one week and FP ~one month) of the different MDs (Table 1). Thus, *K. servazzii* strains were isolated from six firm (3, 5, 7, 9, 11 and 13) and three liquid (2, 6, 8) MDs at BS1 but not at the FP. Strains of *K. bulderi* were isolated from all liquid MDs at FP and *M. guilliermondii* only from liquid MD6, but both species were not isolated from any of the firm MDs. In contrast, *W. anomalus* was isolated only from firm MDs (1, 5, 7, 16) at FP, not from liquid MDs. Moreover, strains of *S. cerevisiae*, *P. fermentans*, and *T. delbrueckii* were isolated from solid and liquid MDs, either at one or during both fermentation steps and depending on flour type: *P. fermentans* from all liquid MDs and the firms except MD9; *T. delbrueckii* from all firm and 5 liquid MDs (2, 4, 10, 12, 14); and *S. cerevisiae* from 11 firm (1, 3, 5, 9, 11, 13, 15, 17, 18, 20, 21) and 3 liquid MDs (2, 4, 14) (Table S3a,b).

From bakery doughs (16 BDs, Table 2), *S. cerevisiae* was the yeast species isolated with the highest frequency (101) and *M. carpophila* strains (2) were isolated from BD8 and BD14 (Table S1).

From raw cereal matrices (Table 3), only one yeast species was frequently isolated (Table S1). *P. fermentans* strains were isolated from barley and oat grains (EE5 and EE9) and from a five-cereal grain mix (EE1). The exception was a type of tritordeum grains (GTit), from which *M. guilliermondii*, *S. cerevisiae*, and *W. anomalus* strains were isolated. From flours (Table S1), *T. delbrueckii* was isolated from wheat, tritordeum, and wholemeal flours; *P. fermentans* from wheat, tritordeum and wholemeal wheat, rye, and tritordeum flours; *W. anomalus* from two types of tritordeum flours; and a strain of *M. carpophila* was isolated from a wholemeal tritordeum flour.

Moreover, a total of 81 isolates were recovered from colonies grown on SDCA plates seeded with samples obtained from cereal grains and flours (79 isolates), from MD19 (1), and from BD3 (1) that correspond to spoilage species (urease positive or that produced filamentous hyphae) and were all discarded (Table S4). Thus, the abundance of spoilage yeast was much greater in raw cereal matrices than in fermented ones, representing ~16% of the total 512 microbial colonies originally selected from SDCA plates at the beginning of this work.

In summary, the diversity of yeast species among the strains isolated from MDs from Spain, made with different flours, is much higher than that found in bakery wheat doughs (BDs). In fact, among the 433 isolates, 8 of 9 yeast species were recovered from 21 MDs, only 2 from 16 BDs, 3 from 10 types of cereal grains, and 5 from 20 types of flours. Thus, 64.35% of the 433 yeasts isolated were recovered from MDs, 28.47% from BDs, 1.62% from cereal grains, and 5.56% from flours.

### 3.3. Metataxonomic Analysis of the Fungal Microbiome in Four Selected Mother Doughs

Indeed, isolation in microbiological cultures may allow the discovery of new yeasts strains useful for the bakery industry and in other food fermentations. However, estimations regarding the prevalence of each yeast species in a given fermented matrix by culture isolation could either reflect its real abundance in the respective matrix or rather its ability to grow on SDCA plates at 28 °C (Section 2.1.1). Therefore, we considered the possibility that a much greater richness of yeast species could exist in these MDs.

To test this idea, we explored the fungal microbiome in selected MDs using a procedure based on Next Generation Sequencing (NGS) developed by Biome Makers (Section 2.3). Amplicon-based metagenomics, also called metabarcoding, rely on the sequencing of the same DNA sequence shared by all the microbes present in a given sample, but also that the DNA sequence is different enough to allow different microorganisms to be identified. For example, for fungi, amplification and sequencing of the 5.8S-ITS1 detects more Operational Taxonomic Units (OTUs) than the D2 region [97].

For a metataxonomic analysis of the fungal microbiome, two MDs (firm MD7 and liquid MD8) made with a wholemeal wheat flour W200 (WMW) and two MDs (firm MD11 and liquid MD12) made with a wholemeal tritordeum flour W100–110 (WMtr) were selected (Table 1). The aim was to compare the yeast species comprising the microbiomes of the different cereal types and MD consistencies and analysis was performed on samples of the first (BS1, ~one week) and final (FP, ~one month) back slopping steps.

**Figure 2:**
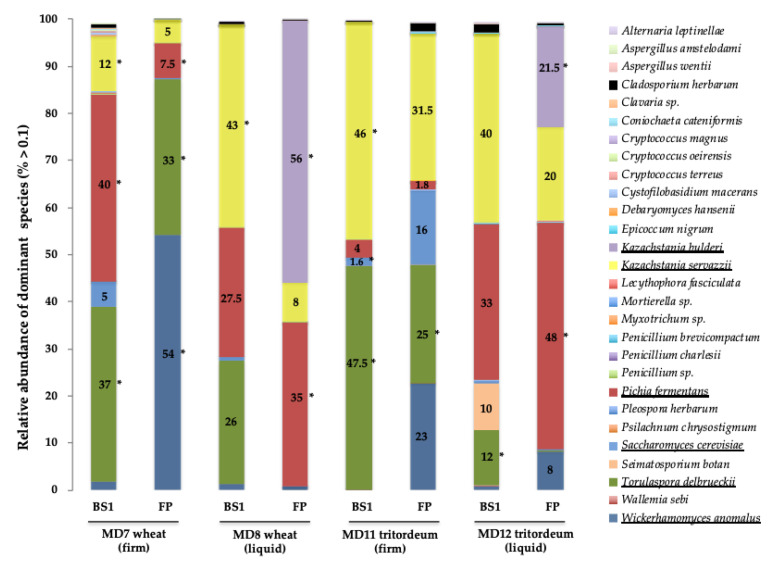
Fungal microbiome of four selected MDs made from wheat (MD7 and MD8) and tritordeum (MD11 and MD12) flours (Table 1); the consistency of the MDs, firm vs. liquid, is indicated. Yeast species with abundance above 0.1% are shown on the right. The species that are underlined are the ones that were isolated using the culture-dependent approach, which are also indicated outside the histogram bars with an asterisk (*) BS1: first back slopping step at optimal fermentative capacity (6–7 days). FP: Final procedure, at the last back slopping step (28–30 days). The relative abundance of yeast species (%) is indicated inside the histogram bars for each MD sample and step. Wholemeal wheat and tritordeum flours were stone milled by “Molinos del Duero” (Zamora, Spain).

The yeast species with a relative abundance above 0.1% found in the four MDs are shown in Figure 2 and the full record of species in the respective fungal microbiomes in Table S5.

In MD7 (made from wheat and of a firm consistency) at BS1, the two dominant species were *P. fermentans* (dark red, 40%) and *T. delbrueckii* (green, 37%), followed by *K. servazzii* (yellow 12%), *S. cerevisia*e (light blue 5%), and *W. anomalus* (dark blue, 1.7%). After ~30 days of back slopping (FP), the dominant species were *W. anomalus* (dark blue, 54%) and *T. delbrueckii* (green, 33%), whereas *P. fermentans* (red, 7.5%) and *K. servazzii* (yellow, 5%) were significantly reduced with respect to BS1, and *S. cerevisia*e remained below the 0.1% threshold of detection (Figure 2).

In contrast, in MD8 (made from wheat and of a liquid consistency), the three dominant species at BS1 were *K. servazzii* (yellow, 43%), *P. fermentans* (dark red, 27.5%), and *T. delbrueckii* (green, 26%). At the FP step, the two dominant species were *K. bulderi* (violet, 56%), even if its abundance was < 0.1% at BS1, and *P. fermentans* (red, 35%), whereas *K. servazzii* (yellow, 8%) was considerably less abundant or displaced relative to BS1. Notably, *W. anomalus* (dark blue) was not dominant in liquid MD8 after one month of back slopping; however, it was dominant in firm MD7 at 1 month, made from the same flour (Figure 2).

In tritordeum firm MD11 at BS1, the two codominant species were *T. delbrueckii* (green, 47.5%) and *K. servazzii* (yellow, 46%), whereas the percentage of abundance of *P. fermentans* (dark red, 4%) and *S. cerevisia*e (light blue, 1.6%) was much lower. At the FP step, the dominant species was still *K. servazzii* (yellow, 31.5%), followed by *T. delbrueckii* (green, 25%), and was reduced by about half with respect to BS1; the relative abundance was similar to that of *W. anomalus* (dark blue, 23%), <0.1% at BS1. Additionally, the relative abundance of *S. cerevisia*e (light blue) was 10 times greater at FP (16%) than at BS1 (1.6%).

In contrast, in tritordeum liquid MD12 at BS1, the two dominant species were *K. servazzii* (yellow, 40%) and *P. fermentans* (dark red, 33%), followed by *T. delbrueckii* (green, 12%), the spoilage yeast *Seimatosporium botan* (light orange, 10%), and *W. anomalus* (dark blue, <0.1%). At the FP step, the dominant species was *P. fermentans* (dark red, 48%) followed by *K. bulderi* (violet, 21.5%) and *K. servazzii* (yellow, 20%) in similar proportions, whereas *W. anomalus* was more abundant (dark blue, 8%) and *S. botan* was completely displaced or undetected.

A quantitative comparison was made between the number of yeasts and the corresponding species isolated on culture plates and the relative abundance estimated by the metataxonomic analysis of the fungal microbiome in the four MD samples (Table 5).

*K. bulderi* isolates (13) were recovered from two liquid MD samples at the FP step (4—MD8; 9—MD12) and was the dominant species (56% and 21.5%, respectively). *K. servazzii* isolates (12) were recovered only from three MD samples at BS1 (1—MD7; 10—MD8; 1—MD11), a result that contradicts what was found in the metagenetic data which indicated its presence in all MD samples (5–46%).

*P. fermentans* isolates (14) were obtained from four MD samples (3, MD7—BS1; 4, MD7—FP; 6, MD8—FP; 1, M12—FP), even though this species was detected in a similar or even greater abundance in other MD samples (Figure 2, Table 5).

*S. cerevisiae* was highly underrepresented in the four MDs analyzed. Only one isolate was recovered from MD11 at BS1 (1.6%) but not from MD11 at the FP, in which its abundance was estimated to be ~10-fold higher (15.7%).

*T. delbrueckii* isolates (32) were obtained from two firm MD samples at both BS1 and FP maturation steps (MD7—BS1 and FP; MD11—BS1 and FP) and one liquid (MD12—BS1) and, although the number of isolates was not proportional to the relative abundance, a significant number of this species’ isolates were recovered from the four MDs.

However, a few isolates of *W. anomalus* (3) were obtained from only one sample (MD7—FP), in which it was the dominant species (54.2%) (Figure 2 and Table 5).

### 3.4. Analysis of Traits of Technological Interest in Selected Yeast Strains

A number of yeast isolates (indicated as selected strains) that represent the species biodiversity found in the matrices of this study and their geographical origin were chosen for further characterization.

#### 3.4.1. *Saccharomyces cerevisiae* Strain Discrimination

**Figure 3:**
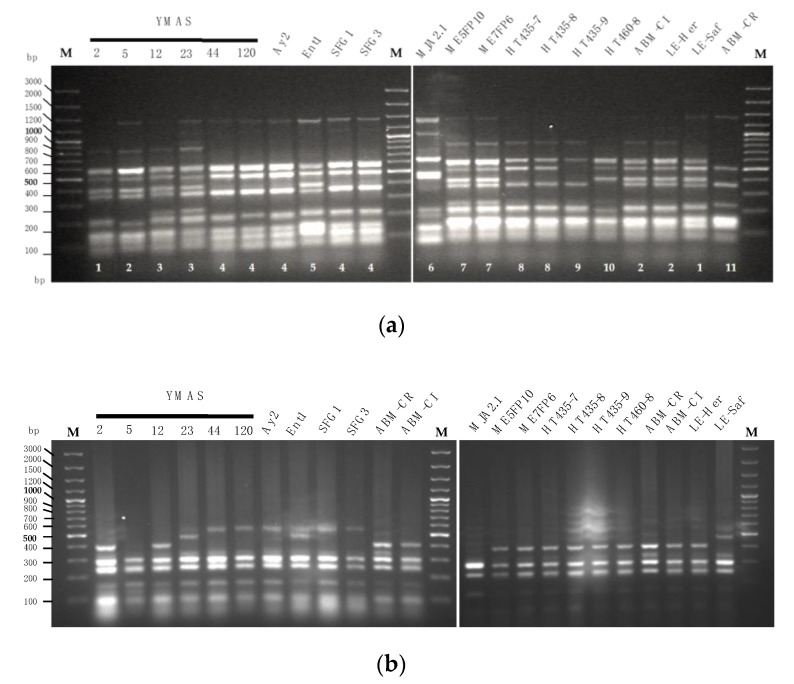

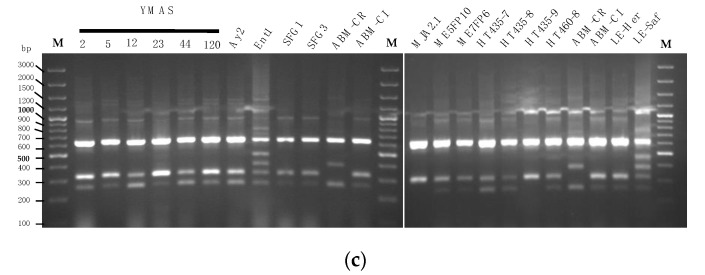
Molecular footprints of selected S*accharomyces cerevisie* strains. (**a**) Inter-δ footprint patterns for 17 *S. cerevisiae* strains: Left panel, 10 strains from BDs; right panel, yeast strains from three MDs (MJA2.1, ME5FP10 and ME7FP6), three novel tritordeum flours (HT strains), two commercial strains from Lesaffre (LE-He, Hércules; LE-Saf; Saf-Instant), and two from AB Mauri Food (ABM-CL, Classic; ABM-CR, Cinta Roja). Numbers in white at the bottom indicate the 11 inter-δ patterns. (**b**) Minisatellite restriction fragment length polymorphisms (RFLP) for genes *AGA1* (*Alu*I) and (**c**) *SED1* (*Hpa*II). M: Molecular weight markers (GeneRuler 100 bp Plus DNA Ladder, Thermo Fisher, Madrid, Spain).

An analysis of polymorphic DNA sequences was performed to discriminate among *S. cerevisiae* strains isolated from MDs, BDs, and commercially available bakers’ yeast strains, as described in the Materials and Methods (2.2.5). The results are shown in Figure 3.

A total of 11 inter-δ footprint patterns were distinguished for 17 *S. cerevisiae* strains (Figure 3a).

Five δ patterns were obtained for 10 *S. cerevisiae* strains isolated from BDs (Table 2) which are shown in Figure 3a (left panel). Pattern #1 was found in YMAS2 (BD2) and the commercial strain LE-Saf (Lesaffre Ibérica, Valladolid, Spain); #2 in YMAS5 (BD2) and two commercial strains, ABM-CL (AB Mauri Food, Córdoba, Spain) and LE-Her (Lesaffre Ibérica, Valladolid, Spain); #3 in YMAS12 (BD2) and YMAS23 (BD7); #4 in five strains isolated from four BDs: YMAS44 (BD10), YMAS120 (BD4), Ay2 (BD13), SFG1 and SFG3 (BD3); #5 was exclusive for Ent1 (BD9) (Figure 3a, left panel).

Six additional δ patterns were distinguished for *S. cerevisiae* strains isolated from three MDs (Table 1) and four new flour varieties of tritordeum and commercial baker’s yeasts (Figure 3a, right panel). Pattern #6 was obtained for MJA2.1 (MD15) and #7 for both ME5FP10 (MD5) and ME7FP6 (MD9) (Figure 3a, right panel).

To test the resolution of the inter-δ analysis, *S. cerevisiae* strains isolated from a different source, the new tritordeum flour varieties HTC435 and HTC460 (Agrasys S.L., Barcelona, Spain), were analyzed. Strains HT435-7 and HT435-8 showed the new pattern #8, HT435-9 for #9, and the HC460-8 for #10. Finally, the commercial ABM-CR strain (AB Mauri, Food Córdoba, Spain) exhibited the unique δ profile #11. These data show that *S. cerevisiae* strains isolated from MDs and tritordeum flours have specific and different inter-δ patterns that likely reflect the different matrix types and/or geographic origin.

The *S. cerevisiae AGA1* and *SED1* genes harbor variable minisatellite-like regions in their coding sequences that may lead to distinct RFLP fingerprints [83]. The *AGA1* minisatellite fingerprints were different for YMAS2 and the baker’s yeast LE-Saf, even if both strains had the same δ pattern #1. YMAS5 had an *AGA1* minisatellite fingerprint different than ABM-CL and LE-Her strains, even if the three strains showed the same δ pattern #2 (Figure 3b). However, besides YMAS2 and YMAS5, neither the *AGA1* nor *SED1* minisatellite fingerprints allowed other *S. cerevisiae* strains with the same inter δ pattern to be distinguished (Figure 3b, c).

#### 3.4.2. Yeast Capacity to Leaven Doughs

**Figure 4:**
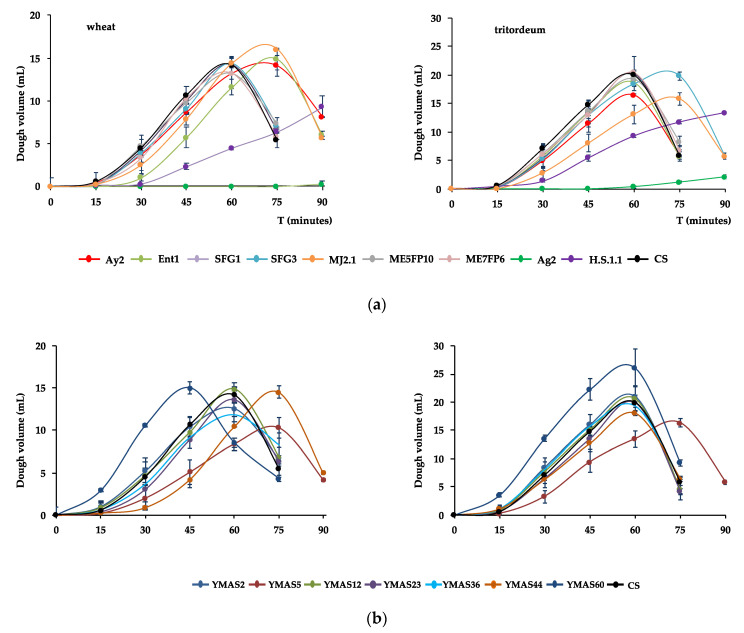
Leavening capacity for 17 yeast strains represented as the increase in dough volume reached at several time points under laboratory conditions (Section 2.4.2). (**a**) Leavening capacity for eight *Saccharomyces cerevisiae* strains from MDs (Ay2, Ent1, SFG1, SFG3, MJA2.1, ME5FP10, and ME7FP6) (Table 1); *Meyerozyma carpophila* Ag2 (BD12, Table 2), *Torulaspora delbrueckii* H.S.1.1 (WMW flour, Table 3), and the commercial baker’s yeast strain CS (ABM-CL). (**b**) Leavening capacity for seven *S. cerevisiae* YMAS strains isolated from BDs and CS. Left panels: wheat; right panels: tritordeum. Strains and sources of origin is shown in Table S1.

The leavening capacity over time (fermentation kinetic) was analyzed for 17 yeast strains in lean doughs after growing the cells in rich YPD medium (Figure 4).

Most of the presumptive wild strains of *S. cerevisiae* were able to leaven plain doughs (no sugar added) made of wheat and tritordeum when tested in pilot assays in graduated tubes (Section 2.4.2). Fermentations were performed in plain doughs made of wheat flour (W~200) from La Vilafranquina (Ávila, Spain) (left panels) and tritordeum flour (W~100–110) from Molinos del Duero (Zamora, Spain) (right panels).

*S. cerevisiae* strains isolated from MDs (Table S1), namely SFG1, SFG3, ME5FP10, and ME7FP6, raised the wheat W200 flour dough as fast as the CS commercial strain (ABM-Classic, AB Mauri Food, Córdoba, Spain), reaching a maximal volume of 13–14 mL at 60 min (Figure 4a, left panel; Table 6). However, *S. cerevisiae* MJA2.1, Ay2, and Ent1 strains raised the wheat dough slower than the previous four strains, reaching a maximal volume similar to CS (~14 mL) or higher (~16 mL, MJA2.1) but more slowly, at 75 min. In contrast, the *T. delbrueckii* H.S.1.1 strain raises the dough much more slowly than the *S. cerevisiae* strains, reaching a maximal volume of only ~9 mL at 90 min. In addition, this strain seemed to continue fermenting beyond this time point. The *M. carpophila* Ag2 strain was unable to ferment the plain wheat dough (Figure 4a, left panel; Table 6).

For tritordeum W100-110 flour, the *S. cerevisiae* SFG3, ME5FP10, and ME7FP6 strains raised the dough as fast as the CS, reaching the maximal volumes of 19–20 mL, which was greater than in wheat dough, except for Ay2 (~16 mL) at 60 min (Figure 4a, right panel; Table 6). In contrast to the leavening capacity in wheat, SFG3 raised the tritordeum dough slower than the CS strain, but reached a similar volume of ~20 mL at 75 min. The MJA2.1 strain also raised tritordeum dough slower than CS and reached a volume similar to Ay2 (Table 6) at 75 min. Finally, *T. delbrueckii* H.S.1.1 raised the tritordeum dough much more slowly than the *S. cerevisiae* strains, and *M. carpophila* Ag2 barely fermented this dough (Figure 4a, right panel; Table 6).

*S. cerevisiae* YMAS2, YMAS12, YMAS23, and YMAS36 strains isolated from bakery doughs (BDs) (Table S1) raised the wheat W200 flour dough as fast as the commercial CS strain, reaching about the same maximal volume at 60 min (Figure 4b, left panel; Table 6). YMAS44 showed a marked lag in the start of fermentation and a slower capacity than CS, but almost reached the same final volume at 75 min. YMAS60 was very fast and reached a volume similar to CS at 45 min, exhibiting the highest fermentation rate among the YMAS strains studied (Table 6). In contrast, YMAS5 had the slowest fermentative kinetic, reaching a volume of only 10.5 mL at 75 min (Figure 4b, left panel; Table 6).

For tritordeum W100–110 flour doughs, the *S. cerevisiae* YMAS2, YMAS12, YMAS23, YMAS36, and YMAS44 strains raised the dough and reached a maximal volume almost identical to the CS strain (Figure 4b, right panel; Table 6). As in wheat flour, YMAS60 raised the tritordeum dough faster and reached a higher volume (21 mL) than CS at the same time point (60 min). YMAS5 raised the tritordeum dough very slowly and reached a lower volume than the other *S. cerevisiae* strains, just as in wheat flour (Figure 4b, right panel; Table 6).

The specific fermentation rates and the maximal volumes reached by each of the 17 yeast strains in each type of dough are shown in Table 6.

These data show that some of the *S. cerevisiae* strains isolated from MDs and BDs have a capacity to leaven lean doughs that is comparable to that of a commercial baker’s yeast CS strain grown in the same manner. The data also suggest that new selected wild strains with fast or slow fermentative kinetics may fulfill the desired prerequisites for some specific technological processes of bread-making.

We also observed that some of the analyzed *S. cerevisiae* strains produced normal colonies of regular size (cells with functional mitochondria) and others as YMAS5 and YMAS23 are *petite* mutants (small-sized colonies, cells unable to respire) that cannot grow on YPG (2% glycerol) plates [98].

#### 3.4.3. Yeast CO_2_ Production

The fermentative capacity was analyzed in Ankom yeast systems (Materials and Methods (Section 2.4.3). In this case, the fermentation of sugars was evaluated in a Model Liquid Dough (MLD) without flour formulated by Panadero et al. [89] and validated by Lesaffre (Marcq-en-Baroeul, France). Thus, the ability to ferment sugars producing CO_2_ was quantified for each strain independently of the type of flour. Cells grown in YPD until the early stationary phase were inoculated in 30 mL of MLD (maltose 4.5% and glucose 1.5%) and the CO_2_ was quantified over the time.

**Figure 5:**
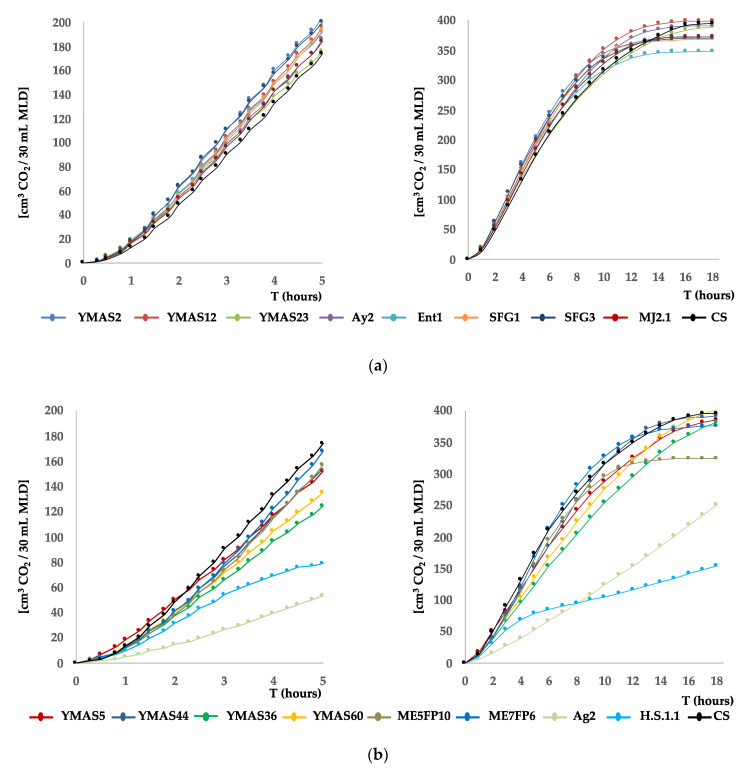
Kinetics of CO_2_ production in Ankom yeast systems (cm^3^ of CO_2_/h) with up to 5 h (left panels) and 18 h (right panels) of fermentation obtained in a representative experiment. (**a**) *Saccharomyces cerevisiae* (YMAS2, YMAS12, YMAS23, Ay2, Ent1, SFG1, SFG3, MJ2.1) and commercial CS strains grown under the same laboratory conditions. (**b**) *S. cerevisiae* (YMAS5, YMAS36, YMAS44, YMAS60, ME5FP10, ME7FP6, and CS), *Meyerozyma carpophila* (Ag2), and *Torulaspora delbrueckii* (H.S.1.1) (Table S1, strain codes and sources).

The kinetics of CO_2_ production for 17 yeast strains are shown in Figure 5 and the respective fermentative rates (cm^3^ of CO_2_/h) and maximal volumes of gas produced are recorded in Table 7.

Some *S. cerevisiae* strains isolated from BDs (YMAS2, YMAS12, YMAS23) and MDs (Ay2, Ent1, SFG1, SFG3, MJA2.1) (Table S1) produced CO_2_ at similar or even faster rates than the commercial CS strain (ABM-Classic) grown under the same laboratory conditions, in higher or similar final amounts (Figure 5a, Table 7). After 5 h of fermentation, the volumes of gas produced by the *petite* YMAS23 and CS strains were similar (~174 cm^3^ CO_2_), or higher for Ay2, Ent1, SFG1, and MJA2.1 (185–196 cm^3^) isolated from MDs (Table S1), and the highest production was observed for SFG3 and YMAS2 (~205 cm^3^) (Figure 5a, left panel; Table 7). The maximal volumes of CO_2_ were reached by YMAS12, Ay2, SFG1, and SFG3 at 10–12 h and by YMAS23 at 16–18 h (Table 7). The exponential production of gas reached a plateau, most likely due to the sugars of the MLD being consumed or exhausted. After 18 h of fermentation, YMAS12, YMAS23, and Ay2 had produced ~400 cm^3^ of CO_2_ similar to CS, but lower amounts were produced by YMAS2, SFG1, SFG3, and MJA2.1 (~370 cm^3^) and Ent1 (~348 cm^3^) (Figure 5a, right panel; Table 7).

Some other *S. cerevisiae* strains isolated from BDs (the *petite* strain YMAS5, and also strains YMAS36, YMAS44, YMAS60) and MDs (ME5FP10, ME7FP6) and two *non-Saccharomyces* strains (H.S.1.1 and Ag2) (Table S1) produced CO_2_ at similar or slower rates than CS, and in similar or lower amounts (Figure 5b, Table 7). After 5 h of fermentation, *S. cerevisiae* strains YMAS36 (150 cm^3^) and YMAS60 (135 cm^3^) produced less gas than CS (~174 cm^3^), and the rest (YMAS5, YMAS44, ME5FP10, ME7FP6) produced less CO_2_ or similar to CS (150–170 cm^3^) (Figure 5b, left panel; Table 7). The slowest and worst CO_2_ producers were H.S.1.1 (*T. delbrueckii,* 79 cm^3^) and Ag2 (*M. carpophila,* 53 cm^3^). After 10 h of fermentation, most of the analyzed *S. cerevisiae* strains reached a maximal volume of CO_2_ similar to that of the CS strain (396 cm^3^), including YMAS36 and YMAS60 that had a slower CO_2_ production rate (Table 7) and with ME5FP10 (324 cm^3^) being the only exception. After 18 h, the lowest amounts of CO_2_ were observed for Ag2 (12 cm^3^) and H.S.1.1 (15 cm^3^) (Figure 5b, right panel; Table 7).

Thus, a number of *S. cerevisiae* strains isolated from BDs and MDs showed CO_2_ production rates in MLD similar to the commercial CS strain (38.7 cm^3^/h), except YMAS36, YMAS60 (28–30 cm^3^/h), and the *non*-*Saccharomyces* Ag2 (12 cm^3^/h) and H.S.1.1 (15 cm^3^/h) strains (Table 7).

#### 3.4.4. Sugar Hydrolytic Activities of *Saccharomyces cerevisiae* Strains

Maltose is the main sugar in wheat and other lean (plain) flour doughs. A high maltase activity has been correlated with the dough leavening abilities of *S. cerevisiae* baker’s yeast strains [99,100] and low maltase activity with the lagging phenotype of some strains in flour fermentations [100].

**Figure 6:**
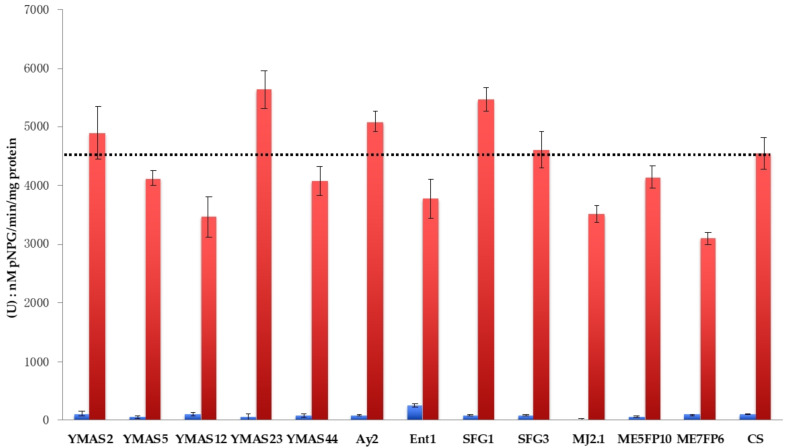
Maltase activity for 12 *Saccharomyces cerevisiae* strains. Cells were cultured under maltase-repressing conditions (glucose, blue bars) and derepressing conditions in maltose containing medium (red bars). The dotted line marks the maltase activity for the reference CS strain (ABM-CL) grown in maltose medium.

Therefore, the maltase enzymatic activity was quantified in 12 presumptively wild *S. cerevisiae* strains from this study and the data of a representative experiment are shown (Figure 6).

*S. cerevisiae* YMAS5, YMAS44, SFG3, and ME5FP10 strains exhibited non-constitutive maltase activities (repressible by glucose) similar to that measured for the CS strain when the cells were grown in maltose (Table 8). The YMAS2 and Ay2 strains had maltase activities slightly higher than the CS strain, whereas SFG1 and the *petite* YMAS23 strain showed the highest maltase values (Table 8). The other five strains showed maltase values lower than CS in maltose. Ent1 had very low but detectable maltase activity in glucose (YPD).

**Figure 7:**
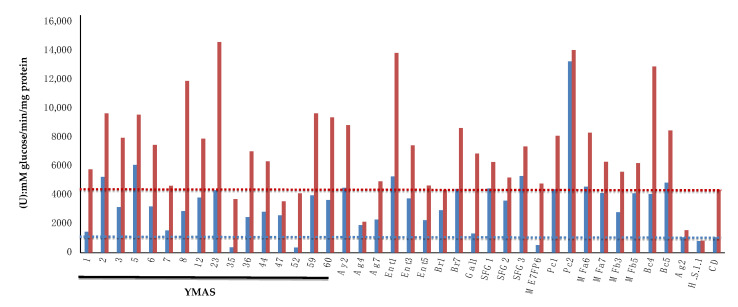
Invertase activity for 3*7 Saccharomyces cerevisiae* strains, *Meyerozyma carpophila* (Ag2), and *Torulaspora delbrueckii* (H.S.1.1) cultured under glucose-repressing conditions in YPD (blue) or in sucrose containing YPS medium (red) obtained in a representative experiment. The dotted lines mark the invertase activity for the CD strain (ABM-CR) under each respective condition.

Sucrose is added to sweet doughs to elaborate some baked goods. A strong correlation was found between the ability o*f S. cerevisiae* to ferment sucrose and the capacity to produce and retain glycerol, which is important for osmoregulation under low water concentrations [56]. Therefore, an initial screening was made to measure the invertase activity for 37 *S. cerevisiae* strains, 2 *non*-*Saccharomyces*, and a CD commercial strain of baker’s yeast recommended for sweet doughs (ABM-CD) (Figure 7; Table 9).

The *S. cerevisiae* strains YMAS8 and *petite* YMAS23 (from BDs) and Ent1, Pc2, and Bc4 (from MDs) exhibited the highest invertase values when grown in sucrose medium (YPS) (Table 9). Interestingly, some *S. cerevisiae* strains showed constitutive invertase activity when grown under enzyme-repressing conditions in YPD (i.e., YMAS2, YMAS5, Ent1, and SFG3, among others), much higher than that of the CD strain (Figure 7). The Pc2 strain isolated from a homemade MD (Dr. PM. Coll, IBFG, Salamanca, Spain) showed a very high invertase activity under both conditions. The Ag2 (*M. carpophila*), H.S.1.1 (*T. delbrueckii*), and Ag4 (*S. cerevisiae*) showed the lowest invertase activities among all strains analyzed (Table 9).

Thus, a number of *S. cerevisiae* strains isolated from MDs and BDs showed invertase activities much higher than that of the CD commercial strain recommended for leavening sweet doughs. Notably, the invertase activity was constitutively derepressed in some of the new strains, with activity values in YPD near to that of the CD strain grown in maltose containing YPS medium.

#### 3.4.5. Screening for Yeast Vitamin Requirement and Riboflavin Production

The requirements of the 433 isolates for vitamins was determined as the ability to grow in a set of supplemented minimal liquid media (Section 2.4.5) and the results are recorded in Table 10. Only the *W. anomalus* isolates were able to grow in a minimal medium without the vitamins tested, and the remaining isolates of other species needed one or more vitamins for growth. Most of the *K. bulderi* (33 of 36), *S. cerevisiae* (157 of 158), and *T. delbrueckii* (73 of 114) isolates, all *K. humilis* isolates (7), a fraction of the *M. guilliermondii* isolates (2 of 10), half of the *M. carpophila*, and a low number of the *P. fermentans* isolates (3 of 62) required biotin. Some isolates of *K. bulderi* (2 of 37), only one of *S. cerevisiae* (1 of 158), most of the *P. fermentans* (56 of 62), and half of the *M. carpophila* (2 of 4) exhibited thiamine auxotrophy. All the *K. servazzii* (22) and a few *P. fermentans* isolates required three vitamins, biotin, thiamine, and niacin.

The 433 yeasts isolated in this work were p-aminobenzoic, folic, pyridoxine, pantothenic, and riboflavin prototrophs, since they are able to grow efficiently in non-supplemented VFMMY and VFMMY+ media (Section 2.4.5).

Among the isolates requiring biotin—*K. bulderi*, *M. carpophila*, and *T. delbrueckii*—some showed a leaky (auxotrophic) requirement for biotin (−/+, Table 10). These isolates were able to grow in the absence of vitamin B_2_, but growth was delayed by 48 h relative to that observed in the same medium supplemented with biotin, which suggests a partial defect in biotin synthesis.

The vitamin requirements of the 433 presumptive wild yeasts strains isolated in this study did not correlate with the fermented or raw matrices from which they were isolated (MD, BM, F, G, Table 8). Thus, isolates from different matrices exhibited auxotrophic requirements for the same vitamin and vice versa; yeasts isolated from the same type of cereal matrix exhibited different requirements. However, *M. carpophila* (H6.1, H6.3) and *P. fermentans* (H5.1, H5.2, H2.1) isolates from flours (Table S1), tritordeum wholemeal flour, and a traditional wheat mix, respectively, were auxotrophic for thiamine. The *S. cerevisiae* isolates from MDs and BDs were all auxotrophic for biotin. The GTi5 from a tritordeum grain (Table S1) required both biotin and thiamine.

**Figure 8:**
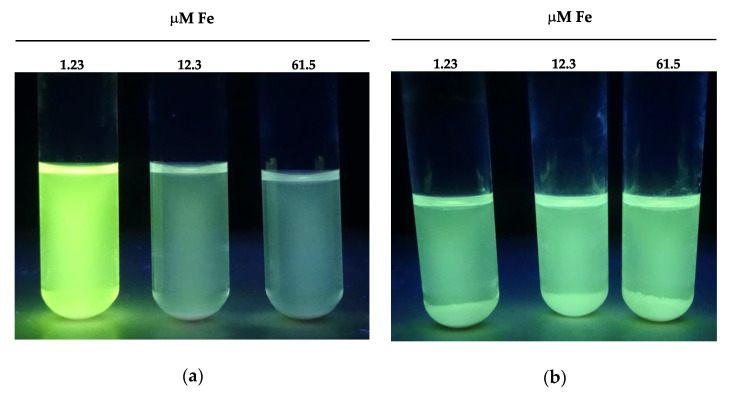
Riboflavin production by *Meyerozyma* species (spp.) on a minimally supplemented medium with increasing concentrations of iron (Fe^+++)^: (**a**) *Meyerozyma guilliermondii* P4A9 strain; (**b**) *Meyerozyma.carpophila* Ag2 strain.

Although the 433 yeasts are riboflavin prototrophs, only *Meyerozyma* spp. exhibited a yellowish fluorescence in the culture medium associated with the overproduction of riboflavin (Figure 8). Riboflavin production by *M. guilliermondii* is strongly influenced by the iron concentration (Fe^+++^) in the culture medium [101]. The production was maximal when iron concentration was low (1.23 μM), reaching a riboflavin concentration of 14.82–16.08 mg/mL in the culture medium (Table 11). By contrast, iron did not affect the production of riboflavin in four *M. carpophila* strains, but its secretion into the culture medium was ~5-fold lower (~4 mg/mL) than that secreted by the *M. guilliermondii* strains (Table 11).

#### 3.4.6. Exocellular Enzymatic Activities of *Non-Saccharomyces* Yeasts

Representative strains of the yeast species isolated from all of the cereal matrices analyzed in this work were tested on culture plates for enzymatic activities that may have a positive influence on dough fermentation (Section 2.4.6) (Table 12). Esterase was detected in all yeasts except for the *P. fermentans* strains, which were positive for protease, gliadinase, and cellobiase activities but not esterase. Protease activity was also detected in the *K. servazzii, M. carpophila*, *M. guilliermondii*, and *W. anomalu*s strains, these being the yeasts with the highest activity halo at 21 mm vs. 15 mm (*P. fermentans*), 7 mm (*M. guilliermondii*), 6 mm (*K. servazzii*), and 5 mm (*M. carpophila*). Gliadinase activity was only detected in the *P. fermentans* and *W. anomalus* strains, which showed a similar halo size (3 mm). Phytase was restricted to yeasts *K. humilis* and *W. anomalus* and cellobiose was detected in all yeast strains, except for *K. bulderi, K. humilis*, and *K. servazzii*. Other enzymes related to the hydrolysis of carbohydrate biopolymers (amylase, β-glucosidase, cellulase, and pectinase) were not detected, although the *W. anomalus* strains were able to hydrolyze pectin (activity halo of 4 mm).

The representative *Saccharomyces* yeast strains ME1A8, M7FP8, YMATi1, P3D10, and P7FP4 from MDs (Table 1, Table S1), YMAS5 and Ent1 from bakery doughs (Table 2, Table S1), and GTit5 from tritordeum grain (Table 3, Table S1) did not exhibit any of the nine exocellular enzymatic activities tested or had activities that went undetected using these assays.

## 4. Discussion

This study is a non-exhaustive overview on yeasts found in a wide variety of fermented and raw cereal matrices originating from Spain. The 67 matrices prospected for new presumptive wild yeasts included 21 mother doughs (MDs), 19 from Spain and 2 from France; 16 bakery doughs (BDs); 20 flours; and 10 types of cereal grains. We relied on a culture-dependent approach to isolate 433 yeasts that were subjected to species identification, which allowed us to select representative strains for further studies and to analyze species biodiversity. We also analyzed the fungal microbiome of four MDs and phenotypic traits of potential technological interest in selected strains.

Identification and species biodiversity analysis are complementary processes which should be combined when large collections of yeast isolates are recovered. The RAPD fingerprinting technique is commonly used to initiate the identification process, as it allows isolate biodiversity to be examined [102,103]. The isolates are then grouped after conducting a mathematical analysis of the RAPD patterns, which facilitates the selection of isolates for genetic analysis. However, since yeast isolates of different genera should not be analyzed together, it becomes necessary to apply a technique to separate them accordingly before analyzing the RAPD patterns. In this work, we achieved this goal by determining the sizes of 5.8S-ITS amplicons, which contain two hypervariable regions that lead to different sizes in different yeast genera [104,105] before the analysis of RAPD patterns, confirming the usefulness of this approach for differentiating isolates from the same and different yeasts species, as was previously reported [40]. The ultra-sequencing of the 5.8S-ITS amplicon is commonly used in metagenetic techniques of fungal population analysis [44,104,105].

Yeast identification at the genus and species levels is currently based on the sequences of the D1/D2 domain of the large subunit region of the 28S rRNA gene (LSU) and the 5.8S-ITS (Internal Transcribed Sequence), located between the 18S rRNA and 28S rRNA genes [106]. The threshold values were established as 98.41 % for 5.8S-ITS and 99.51 % for the D1/D2 domain of the LSU for species differentiation [104]. Similar or higher values found for both regions allowed the identification of the 433 yeasts isolated in this study as *K. bulderi*, *K. humilis*, *K. servazzii, M. guilliermondii*, *M. carpophila*, *P. fermentans*, *S. cerevisiae*, *T. delbrueckii*, and *W. anomalus*, all belonging to the order *Saccharomycetales* (Table 4 and Table S2).

*Saccharomycetales* species involved in bread-related fermentation are most often the only species found using culture-based methods [30]. The prospective analysis of a wide range of cereal matrices from Spain allowed us to identify yeast species with the status of Qualified Presumption of Safety (QPS), which are considered as food-grade microorganisms [107,108,109]. Of the 513 microbial colonies originally recovered from different matrices, 433 isolates belonging to 9-QPS yeast species were selected for further studies (Table S1) and 81 were discarded (~16%) (Table S4). Therefore, some of the yeast isolates characterized in this study may represent novel strains that could be considered for developing new starters for bread-making or other food fermentations [110].

### 4.1. Species Biodiversity in 433 Yeasts Isolated from 67 Cereal Matrices

Remarkably, 299 of the 433 yeasts isolated in this study were recovered from 21 MDs (69%).

*S. cerevisiae* was the yeast most frequently isolated (37% of 433 yeasts, Figure 1a). Notably, 57 of the 159 isolates of this species were recovered from MDs (Figure 1b, Table S3) and 101 from bakery doughs (Figure 1c), as reported for sourdoughs made in other European countries [30,31,40,42,43,44,111,112]. *S. cerevisiae* is the yeast most reported to be found in wheat and rye sourdoughs [25,112,113] and in bakery doughs [29,40,111,114,115,116] most likely due to the contamination of baker’s yeasts [29]. *S. cerevisiae* is a generalist species with the capacity to thrive in a wide range of microbial ecosystems. Accordingly, it was the most common species found in 353 sourdoughs recorded from 1971 [4] and in sourdoughs from around the world (http://robdunnlab.com/projects/sourdough/map/ accessed on 24 December 2020).

Given the influence of environment on the microbial community composition and dynamics of different sourdoughs, laboratory and bakery production conditions need to be tested for microbial species diversity [4,18,47,112,116,117,118]. In this study, 14 MDs were not started or propagated in a laboratory. Instead, all of them were carefully prepared and back slopped by the same expert baker under controlled conditions using a variety of flours and the same type of filtered tap water. Therefore, at least some of the 38 *S. cerevisiae* isolates from 8 of the 14 MDs (Table S3a) and 19 isolates from 5 MDs collected elsewhere (Table S3b) may correspond to new, wild strains of this species.

To test this idea, four *S. cerevisiae* isolates recovered from MDs and four commercial baker’s yeast commonly used in Spain were characterized to the strain level (Figure 3). Distinct δ profiles are likely related to independent mobility events of Ty transposons in the genome of each strain, their number, and/or the recombination among directly repeated delta-LTR sequences, leading to polymorphic DNA patterns [82]. The δ pattern of MJA2.1 (MD15) was clearly different to those of commercial baker’s yeasts (Figure 3a, right panel), suggesting that it could be a new and wild strain. In contrast, the δ pattern for two MD isolates (MD4 and MD6) and the minisatellite fingerprints (Figure 3b,c) resemble those of commercial strains. We conclude that the δ profiles better reflect the different matrix and geographical origin of *S. cerevisiae* isolates obtained from MDs.

The second most frequently isolated yeast species was *T. delbrueckii* (26% of 433, Figure 1a). However, it cannot be discarded that this species may have originated from the bakery food chain, because 109 of the total 114 isolates were recovered from 12 of the 14 MDs made with 8 different flours by the same baker (Table S3a). In fact, the microbial species diversity of the sourdoughs seems to be influenced by the house microbiota of the producer [47]. *T. delbrueckii* has been isolated from several sourdoughs—Austrian and Italian [31,40], firm and liquid [119], and of the Black Sea region [120].

Other yeast species were recovered with lower frequency from MDs of this study than the two mentioned above. The third yeast most frequently isolated was *P. fermentans* (14%, Figure 1a) and 53 of the total 62 isolates were also recovered from 14 MDs made for this work (Table S3a). *T. delbrueckii* and *P. fermentans* were found with *S. cerevisiae* in spelt sourdoughs [20].

The fourth group of yeasts belongs to three species in the *Kazachstania* clade*—K. bulderi* (8%), *K. servazzii* (5%), and *K. humilis* (2%) (together representing the 15% of the 433)—and 64 of the 65 isolates of this genus were recovered from MDs (Table S3). Several *Kazachstania* spp. were isolated from sourdoughs and also from the bakery environment [24,25,27,29,112,119] and were dominant in organic French sourdoughs [30]. In this work, *K. humilis* was isolated only from MD18 and MD19 (Table 1), which is the dominant species in some French sourdoughs [27] and was also found in certain Italian sourdoughs [119]. *K. humilis* and *S. cerevisiae* were the most abundant species found in Belgian sourdoughs [24]. In this study, *K. servazzii* and *K. bulderi* were isolated only from MDs made by the same baker, *K. servazzii* from firm and liquid MDs, and *K. bulderi* from seven liquid MDs (Table S3a). This result suggests that these two species could have their origin from the bakery environment or, more unlikely, would be present in eight different flours. *K. servazzii* has been found in Italian [119] and French sourdoughs [26]. Recently, *K. bulderi* and *K. servazzii* were isolated with *P. fermentans* from Turkish sourdoughs [120]. *K. bulderi* was only described in sourdoughs from France [3]; therefore, this study would be the first report identifying this species in MDs made in Spain.

Among the less frequently isolated species, *W. anomalus* was isolated from 4 of the 21 MDs (3%, Figure 1b), a generalist species found in some Belgian sourdoughs [42,117] but not in France or Italy [3]. Finally, *M. guilliermondii* was isolated only from MD6 (Table S3a). This species was found in fermented doughs prepared in Spain using apples, whereas *S. cerevisiae* was the dominant species, with a lower proportion o*f M. guilliermondii* and *W. anomalus* in those made with yogurt [43].

In this study, only one yeast species was most frequently isolated from 10 of 21 MDs at any given time (MDs 8, 9, 10, 11, 12, 15, 16, 17, 19, 20, 21), which may correspond or not to the most abundant species in each MD (Table S3). We propose that inter-yeast communities may exist in the 14 MDs made for this study: the four most frequently isolated species (*T. delbrueckii, S. cerevisiae*, *K. servazzii, P. fermentans*) may establish symbiotic or mutualistic interactions among them and other accompanying species (*W. anomalus*, *K. bulderi*, *M. guilliermondii*, or *P. fermentans*) (Table S3a). In contrast, in seven MDs collected elsewhere (Table S3b), *S. cerevisiae* (MDs 15, 20, 21) and *K. humilis* (MD19) were the only isolated species, as described for Belgian sourdoughs [24].

Recently, the microbial ecology of sourdoughs has been reviewed with a focus on yeasts and the most prevalent species have been determined to be *K. humilis*, other species of the *Kazachstania* clade, and *S. cerevisiae* [4,24,30,47,121,122,123]. Moreover, S*. cerevisiae, W. anomalus, T. delbrueckii, P. kudriavzevii, K. exigua*, and *K. humilis* are the most geographically widespread species in sourdoughs [3] (http://robdunnlab.com/projects/sourdough/map accessed on 24 December 2020).

Therefore, most of the yeast species found in sourdoughs worldwide have also been found in MDs analyzed in this study, with the exception of *P. kudriavzevii* and *K. exigua.*

Cereal grains and flours can be sources of wild yeasts that may have a strong impact on the establishment of stable yeast associations in the sourdoughs [29,36,39,124]. Consequently, the diversity of yeasts found in sourdoughs made and propagated under identical conditions with the same flour as the only non-aseptic material would include at least some of the same species. This could possibly be the case of *T. delbrueckii*, found in wheat flours (H1, H2, and H3) and in firm and liquid MDs (1, 2, 3, 4, and 7) made using these flours. Other yeast species were also found in flours (Table 3) and MDs made with these flours (Table 1), as is the case for *P. fermentans* (flour H5 and MDs 5 and 6; flour H2 and MDs 7 and 8); *K. servazzii* (flour H4 and MD14); or *W. anomalus* (flour T9 and MD16). Interestingly, *Meyerozyma* spp. and *M. carpophila* found in WM tritordeum flour H6 and *M. guilliermondii* in tritordeum grain GTit have never been reported to be present in flours or cereal grains. Yeasts can also be present in mills, bakery rooms, workers’ hands, air, or water [17].

Studies on the diversity of yeasts in raw cereal materials are still scarce. Yeasts found in cereal grains and flours include, among other species, *Torulaspora* spp. and *W. anomalus* [124]. However, the compilation of yeast species diversity in laboratory sourdoughs made under aseptic conditions suggested an autochthonous flour origin of *Kazachstania unispora*, *K. humilis*, and even *S. cerevisiae* [25]. Consistent with those reports, *W. anomalus*, *T. delbrueckii*, and *P. fermentans* were the most frequently isolated species from the flour samples of this study and *K. servazzii* from some of them (Figure 1d). *W. anomalus* and *P. fermentans* were also the species most frequently isolated from grains (Figure 1e). *Meyerozyma* spp. have been isolated from fermented pineapple pulp [125] and from laboratory sourdoughs prepared with fermented apple juice [43]. Antifungal activity has been found in fermented doughs using selected *M. guilliermondii* strains [76]. However, *S. cerevisiae* was never isolated from the 20 types of flours prospected in this study (Figure 1d), although it was later isolated from HTC flours of tritordeum (Figure 3); in fact, it was suggested that this species may have a flour origin [111,112,126]. *S. cerevisiae* was also isolated from tritordeum grains (GTit), a species not reported to be found in cereal grains. A possible association of *M. guilliermondii*, *M. carpophila*, and *S. cerevisiae* species with tritordeum raw matrices should require further research.

Therefore, a broad diversity of yeast species has been isolated from 21 MDs, 19 of them made or collected in Spain, and from raw cereal materials. Thus, of the eight species found in MDs (*K. bulderi*, *K. humilis*, *K. servazzii*, *M. guilliermondii*, *P. fermentans*, *S. cerevisiae*, *T. delbrueckii*, and *W. anomalus*), four were also isolated from flours (*K. servazzii*, *P. fermentans, T. delbrueckii*, and *W. anomalus)* and four from cereal grains (*M. guilliermondii*, *P. fermentans*, *S. cerevisiae* and *W. anomalus*), suggesting that at least some yeasts species found in MDs could have their origin in grains and in the flours used to make them.

Unlike what was observed in MDs, the 16 BDs were uniform in terms of yeast species diversity. The repeated permanence of *S. cerevisiae* in Spanish BDs (101 isolates, Figure 1c and Table S1) could be due to the use and/or presence of baker’s yeast in the bakery environment, as proposed by other authors [29,112]. However, different δ and/or minisatellite patterns were obtained for *S. cerevisiae* strains isolated from bakery doughs, like YMAS2, YMAS5 (BD1), and Ent1 (BD9). Subsequently, at least some *S. cerevisiae* strains isolated from BDs of Spain would correspond to new strains. The occurrence of *S. cerevisiae* and *M. guilliermondii* has been previously reported in some Spanish doughs [32,33]. In this study, *M. carpophila* was isolated only from BD8 and BD14 (Table 2), a species isolated from bakery doughs but not sourdoughs [27] suggesting that the best ecological niche for this yeast is the bakery environment.

In summary, the yeast species most frequently isolated from 67 cereal matrices in Spain were 8 from MDs, only 2 from BDs, 5 from flours, and 4 from grains. Four species were isolated from grains (*M. guilliermondii*, *P. fermentans*, *S. cerevisiae*, and *W. anomalus*) of which two have also been found in flours (*P. fermentans*, *W. anomalus*) but not the other two (*M. guilliermondii* and *S. cerevisiae*). Of five species found in flours, *M. carpophila* appears in BDs and the other four (*K. servazzii*, *P. fermentans*, *T. delbrueckii*, and *W. anomalus)* do not. From sourdoughs, species found in flours (*K. servazzii*, *P. fermentans*, *T. delbrueckii*, *W. anomalus*) or grains (*M. guilliermondii*, *S. cerevisiae*) were found, but others could come from the bakery environments (*K. bulderi*, *K. humilis*).

Metagenetic analysis in a greater number of MDs, BDs, and raw matrices may shed light on the wealth of yeast species present in each matrix, and those that may come from bakery rooms.

### 4.2. Influence of the Flour, Consistency, and Age on the Yeast Species Found in MDs

Regarding the type of flour, both high and low extraction rate flours of wheat and tritordeum were used in 14 MDs of this study (Table 1). The high content of fiber and bioactive compounds in bran may have an impact on the microbial diversity of sourdoughs [37,127,128]. However, a correlation between the number of yeast species isolated from a given MD and the respective flour extraction rate has not been observed in this study (Tables S1 and S3).

Regarding mother dough consistency, very few studies have addressed the microbial, chemical, and technological changes that occur in switching from firm to liquid sourdough fermentation. However, many bakeries have chosen the use of liquid instead of firm sourdough, because it does not seem to affect the typical nature of their products and is much easier to use [119,128,129]. Some authors have found less microbial diversity in liquid than in firm sourdoughs, although the proportion of yeast cells relative to LAB was higher in liquid sourdoughs [119]. Based on our isolation data, the number of yeasts species seems to be greater in firm than in liquid MDs made with the same flour at one week of back slopping, but not after one month (Table S3a). Perhaps, the low buffering capacity and a high rate of acidification could lead to the prevalence of more acid-tolerant yeasts in liquid sourdoughs [24]. Certainly, the number of *T. delbrueckii* and/or *K. servazzii* isolates was greater from liquid than from solid MDs at one week; in addition, most of the isolates of *K. bulderi* and/or *P. fermentans* were recovered from the seven liquid MDs after one month (Table S3a), suggesting that these latter yeasts may be more acid-tolerant species in liquid MDs.

Unlike what happens in liquid sourdoughs, it has been described that the concentration of acetic acid and even of lactic acid increases through propagation in firm sourdoughs (42), which could be related to the disappearance of *K. servazzii* and the isolation of *W. anomalus* from some firm MDs after one month (Table S3a). Although the tolerance of *K. servazzii* to these acids is unknown, *W. anomalus* can use lactic acid in sourdoughs [25]. Nevertheless, the relevance of changes observed in yeast species isolated from firm and liquid MDs would require further research.

### 4.3. Metataxonomic Analysis of Yeast Communities in Four Selected MDs

Culture-independent methods based on high-throughput sequencing technologies are widely used in food microbiology and have recently been applied to analyze the taxonomic structure of sourdoughs [46,47]. We used a metagenetic technique to investigate the structure of the fungal microbiome in four selected MDs made with wholemeal flours of wheat and tritordeum (Table S5). The metataxonomic analysis revealed a great richness of fungal species in the microbiome of the four MDs.

The number of fungal species seems to be greater in the two liquid than in the two firm MDs, and in tritordeum than in wheat at one month; the number of fungal species was reduced over the time in wheat, whereas it increased in tritordeum MDs (Table S5).

Our analysis showed the influence of the flour and the dough consistency in the early and late imposition of yeast species in the different MDs (Figure 2, Table 5). Different species ratios may also reflect the influence of back slopping in the four MDs. Thus, *T. delbrueckii* remained in firm MDs of wheat and tritordeum at one month (reduced abundance) and *P. fermentans* in liquid MDs of both cereals (increased abundance); similarly, *W. anomalus* was present in the firm MDs of the two cereals, whereas *K. bulderi* appeared in the liquid MDs. The increase in initially nonabundant, or almost undetectable, yeast species (*W. anomalus, K. bulderi, S. cerevisiae*) may be favored by changes over time in the intrinsic properties of the MDs (nutrients, pH, acids) and/or the presence of different LAB species not reported in this work (our unpublished data).

The large number of 57 *S. cerevisiae* isolates recovered from 21 MDs may not necessarily reflect the abundance of this species. Our data (Figure 2 and Table S5) indicate that *S. cerevisiae* was either absent or present at very low abundance in the microbiome of four MDs, except in tritordeum MD11 at one month (~16%). Other species were unevenly isolated from the four MDs: *T. delbrueckii* from MD12 (~12%) but not from MD8 at one week (~26%); *P. fermentans* only once and from MD12 (~48%); in contrast, *K. bulderi* was isolated from liquid MDs of the two cereals and *K. servazzii* was isolated from wheat MD8 but not from tritordeum MD12, in which its relative abundance was similar or even higher. By contrast, *W. anomalous* was not isolated from MD11 at one month (~23%). These data suggest that biased interpretations of the richness of yeast species in MDs could be due to the better or worse ability of the respective species/strains to grow on SDCA plates and/or to the isolation procedures used in this work.

Taking together, the data on the fungal microbiome of 4 of the 21 MDs made by the same baker highlight the influence of the cereal flour, consistency, and age, as well as the influence of the bakery environment on the dominance and relative abundance of different yeast species in the MDs. Our results corroborate the importance of using combined analytical approaches to explore the yeast communities of the sourdoughs and suggest there is a need to use a broader group of culture media and conditions as a means to isolate a wider range of yeast species and novel strains from worldwide sourdoughs [31,46,47].

### 4.4. Phenotypic Analysis of Selected Yeast Strains

S*. cerevisiae* is the most commonly used species in starters for bread-making, and the technological features of new strains include a fast carbohydrate fermentation rate and high dough leavening ability [130]. In addition to sugars naturally present in flours, maltose released by the amylolytic breakdown of the starch is the main sugar available to be fermented by yeasts in lean doughs, and the ability to ferment maltose is directly linked to the fermentation performance [55,100]

Therefore, we evaluated some of these technological parameters in sets of *S. cerevisiae* strains selected from the 158 isolates included in this study, namely: (i) the dough-leavening ability in wheat and tritordeum pilot doughs; (ii) the CO_2_ production rates in a model of lean dough without any flour (MLD); and (iii) the maltase and invertase enzymatic activities to evaluate the maltose and sucrose utilizing capacity of several strains.

We selected 17 strains based on the results of the pilot dough fermentations of wheat and tritordeum flours. Thus, SFG3, ME5FP10, and ME7FP6 strains of *S. cerevisiae* isolated from MDs and YMAS2, YMAS12, YMAS23, and YMAS36 strains isolated from BDs raised the wheat and tritordeum doughs almost as fast as the commercial strain CS and some of them reached similar final volumes at about the same time or not much later. The YMAS60 strain is one of the best performing strains relative to the two parameters and in both flours (Figure 4, Table 6). However, novel strains are also desired with slower fermentation kinetics than those of the commercial starters (i.e., *S. cerevisiae* MJA2.1, Ay2, Ent1, YMAS44, and YMAS5 or *T. delbrueckii* H.S.1.1) for the industrial production of frozen breads (Atrian Bakers, Barcelona, Spain, personal communication).

Using a liquid model of lean dough (MLD) without flour, with sorbitol as the osmotic stabilizer and fermentable carbohydrates [89]*. cerevisiae* strains isolated from MDs and BDs showed, in general, CO_2_ production rates similar or slightly higher than the commercial CS strain (Table 7). In accordance with this finding, wild strains isolated from sourdoughs were reported to have higher leavening power than commercial baking yeasts [130]. In the MLD system, the CO_2_ production rate depends on the respective ability of the yeast strains to ferment glucose and maltose under specific osmolarity conditions.

*S. cerevisiae* strains that rapidly utilize maltose and are tolerant to high levels of sucrose are desired for the bakery industry [131]. In fact, genetically modified strains with these two features were proposed in the past for commercial use [132]. We isolated in this study strains of *S. cerevisiae* that exhibit high levels of maltase and invertase activities. Thus, SFG1 and the *petite* YMAS23 strain exhibit the highest maltase activity values in the presence of maltose among the 17 analyzed strains, and Ent1 had low but detectable maltase activity even under glucose repressive conditions (Figure 6). Moreover, YMAS8, YMAS23, and Ent1 isolated from BDs and the Bc4 strain obtained from MDs exhibited much higher invertase values when grown in sucrose medium than the commercial baker’s yeast of reference ABM-CR (Figure 7). Notably, the *petite* YMAS23 strain exhibits high maltose and invertase activities. Moreover, YMAS2, YMAS5, Ent1, and SFG3 showed high and also constitutive invertase activity when grown in glucose, as well as the household isolated Pc2 strain (Table 9). The invertase activity of *S. cerevisiae* results in partial hydrolysis of flour fructans and may contribute to the reduction in FODMAPS in bread [133,134,135].

We conclude that a considerable number of presumptive and wild *S. cerevisiae* strains isolated and phenotypically characterized in this study have properties of interest to be analyzed further and tested in the development of modern, inoculated sourdoughs and the production of pilot breads. Moreover, because some *S. cerevisiae* strains produce secondary metabolites, inhibit the growth of aflatoxin-producing molds, and may have lipolytic, proteolytic, pectinolytic, or glycosidase enzymatic activities, evaluation of these traits in the new strains would require further studies [136].

Yeasts can synthesize vitamins that can be supplied to the diet through bread, although the growth of some strains and yeast species require specific vitamins to grow [137]. In this study, 433 yeasts produced five out of the eight B group vitamins (B_2_, B_5_, B_6_, B_9_, and B_10_), but ~84% display some vitamin requirements. *T. delbrueckii* (37) and *M. guilliermondii* (8) represent 10% of the total yeasts and do not strictly require vitamins to grow, although a 24 h lag phase was observed in the absence of biotin. Growth in a vitamin-free medium is compromised for all of the *W. anomalus* isolates and four of the *T. delbrueckii* isolates. Three patterns of vitamin requirements were found for biotin (64% of 433 strains), thiamine (14.3%), and biotin–thiamine–niacin (5.7%), indicating that leaky or full vitamin requirements are not species-specific but rather strain-dependent.

The autonomy of yeasts to biosynthesize vitamins can be considered in order to formulate mixed yeast starters for bread fermentations, as occurs for vitamin D [61] or B_9_ [60]*. M. guilliermondii* and *M. carpophila* are two close phylogenetically related yeast species [138]; *M. guilliermondii* produces riboflavin (B_2_) depending on the iron concentration in the medium [101], whereas reports for *M. carpophila* have not been found. We found that two *M. carpophila* strains overproduce B_2_ in an iron-independent manner, although to a 4-fold lesser extent than the ten *M. guilliermondii* strains isolated in this work (Table 11). The iron independent B_2_ production of *M. carpophila* could be exploited in fermentations of cereal matrices with as high iron concentrations as some wheat flours [139]. Notably, the two *M. carpophila* strains produce higher amounts of B_2_ (~4000 μg/L) than the best *Lactobacillus fermentum* strain reported for B_2_-enriched breads, which produces 1500-fold less (~1203 μg/L) [140], a species renamed as *Limosilactobacillus fermentum* [141]. A strain of *L. plantarum* was described that produces B_2_ in amounts similar to our *M. carpophila* strains, now renamed as *Lactiplantibacillus plantarum* [142]. The iron content in wheat flour can be up to 15-fold higher [139] than the concentration that inhibits B_2_ production by *M. guilliermondii* strains (~12.3 µM), suggesting the possible utilization of *M. carpophila* to enrich the B_2_ vitamin content of bakery products, instead of or together with *L. plantarum* [142].

During food fermentation, microorganisms produce enzymes to break down polymeric and complex compounds to simple biomolecules for several biological activities such as proteinases or amylases. Among the 433 yeasts, 23 *non-Saccharomyces* isolates were selected as representative from different matrices to evaluate the production of 9 extracellular enzymatic activities (Table 12). All isolates of the same species exhibited the same enzymatic activities, whereas the number of detected activities largely varies among different species. The 23 non-*Saccharomyces* selected strains displayed at least 1 in 9 of the extracellular enzyme activities tested, which may contribute to provide some specific features to bread associated with sourdough fermentations [9]. Esterase could contribute to sourdough flavor complexity as described for wines [143,144,145]. The protease and gliadinase activities of the *P. fermentans* and *W. anomalus* strains may contribute to the detoxification of glyadins [10].

In addition, the protease activities of *K. servazzii*, *M. carpophila*, *M. guilliermondii, P. fermentans*, and *W. anomalus* may contribute to the increase in peptides and free amino acid content of the sourdoughs, and the phytase activities of the *K. humilis* and *W. anomalus* to reduce the phytate content of wholemeal flours [144]. However, although β-glucosidase is commonly detected in *W. anomalus,* non-*Saccharomyces*, and sometimes in *S. cerevisiae* strains, it has not been detected in the yeasts analyzed in this work [146]. Other authors have not detected this activity in specific strains of *W. anomalus* or non-*Saccharomyces* yeasts [95], suggesting the β-glucosidase may be strain-dependent, and/or dependent on the source of origin. The cellobiase activities of *M. carpophila, M. guilliermondii, P. fermentans, T. delbrueckii*, and *W. anomalus* may break down the disaccharide cellobiose, which could be produced from the hydrolysis of the cellulose in wholemeal flours, contributing to reduce the FODMAP in breads fermented with sourdoughs containing these strains [12].

Therefore, we identified yeast species and strains capable of overproducing B_2_ or exocellular enzymes that may contribute to confer better nutritional quality and digestibility to inoculated mother doughs and to new baked goods.

## 5. Conclusions

This work is the first comprehensive study on yeasts found in a wide number of raw and fermented cereal matrices originating from Spain and provides information on specific strains with phenotypic traits of biotechnological interest. The Spanish PANLEV collection is worthy of being preserved, as it contains a high biodiversity of yeasts that could be exploited for technological applications in the food field.

Among the wide repertoire of 433 yeast isolates of 9 species, some strains showed relevant traits for potential applications in bread-making. *S. cerevisiae* strains that exhibit high levels of maltase and invertase activities and CO_2_ production could be useful for efficient fermentations of wheat and/or tritordeum flours. Strains of two *Meyerozyma* spp. that overproduce vitamin B_2_ may increase the nutritional value of doughs inoculated with them and of the breads. Enzymatic activities detected in strains of *W. anomalus* and *P. fermentans* may contribute to a better digestibility of wheat breads and phytase activity detected in some *W. anomalus* or *K. humilis* strains suggests a possible use for fermentations of wholemeal wheat flours.

The novel yeast strains isolated and characterized in this study may fulfil at least some of the common requirements to formulate new, single, or mixed yeast starters, alone or in combination with lactic acid bacteria, to improve the microbial safety of modern inoculated sourdoughs and elaborate new breads with distinctive organoleptic, sensory, and nutritional profiles.

## Figures and Tables

**Table 1:** Type I mother doughs (MDs) used for the isolation of yeasts.

MD#	Consistency	Flour Type	Baker (Location)
MD1	Firm	wheat, W180–200 ^(a)^	EM (Zamora, Spain)
MD2	Liquid	wheat, W180–200 ^(a)^	EM (Zamora, Spain)
MD3	Firm	wheat, W130–150 ^(a)^	EM (Zamora, Spain)
MD4	Liquid	wheat, W130–150 ^(a)^	EM (Zamora, Spain)
MD5	Firm	T. Zamorana W230–250 (TZM) ^(a)^	EM (Zamora, Spain)
MD6	Liquid	T. Zamorana W230–250 (TZM) ^(a)^	EM (Zamora, Spain)
MD7	Firm	wholemeal wheat (WMW) ^(a)^	EM (Zamora, Spain)
MD8	Liquid	wholemeal wheat (WMW) ^(a)^	EM (Zamora, Spain)
MD9	Firm	tritordeum W100–110 (tr) ^(a)^	EM (Zamora, Spain)
MD10	Liquid	tritordeum W100–110 (tr) ^(a)^	EM (Zamora, Spain)
MD11	Firm	wholemeal tritordeum (WMtr) ^(a)^	EM (Zamora, Spain)
MD12	Liquid	wholemeal tritordeum (WMtr) ^(a)^	EM (Zamora, Spain)
MD13	Firm	wheat “Oromas” ^(b)^	EM (Zamora, Spain)
MD14	Liquid	mixture of 6 flours (MO6F) ^(a)^	EM (Zamora, Spain)
MD15	Firm	wheat ^(d)^	MJA (Valladolid, Spain)
MD16	Firm	tritordeum W100–110 (Tr) ^(c)^	MJA (Valladolid, Spain)
MD17	Firm	wholemeal tritordeum (WMtr) ^(a)^	MJA (Valladolid, Spain)
MD18	Firm	tritordeum W100–110 (tr) ^(c)^	JAR (Barcelona, Spain)
MD19	Firm	wheat “T-80” W200–220 ^(c)^	JAR (Barcelona, Spain)
MD20	Firm	wheat ^(d)^	TB (Boulogne-sur-Mer, France)
MD21	Firm	wheat ^(d)^	FB (Boulogne-sur-Mer, France)

MDs numbered 1–14 were started and propagated for this work by the baker E. Mateos (EM), Reposteria Mateos S.A—Pan del Duero—Mahorpan (Zamora, Spain), using the flours indicated; MDs 15–17 were homemade by M.J. Asensio (MJA); MDs 18–19 by baker J.A. Ribas (JAR, Forn Cruixent (Barcelona, Spain); MDs 20–21 were brought from Boulogne-sur-Mer (France) by M. Sánchez (Confitería Santa Lucía, Villares de la Reina, Salamanca, Spain) and made by a travelling baker (TB) and Fred Bakeries (FB). The consistency of the MDs by approximate dough yield (DY) is indicated as firm or liquid. The specific strength of the flour (W) is indicated when known. Wholemeal flours are indicated (WM) and all others were refined flours. (a) Ecological grade flours from Carbajo Hermanos, S.A. (Molinos del Duero, Zamora, Spain). MO6F is a mixture of 6 flours (wheat flours W200 and W130; a mixture of wheat flours, “Tradicional Zamorana” (TZM); wheat WMW and tritordeum Tr and WMTr). (b) The wheat flour “Oromas” was from La Vilafranquina (Ávila, Spain). (c) Ecological grade tritordeum W100–110 and wheat W200–220 “T-80” flours were stone milled in Farinera Roca S.A. (Lleida, Spain). (d) Wheat flours of unknown features and origin.

**Table 2:** Bakery doughs (BDs) from Castilla y León used for the isolation of yeasts.

Province	BD#	Bakery or Baker (Town)
Salamanca	BD1	A. Sánchez (Cabrerizos)
	BD2	San Morales (Cabrerizos)
	BD3	V. Hernández (San Felices de los Gallegos)
	BD4	L.E. Río (Lumbrales)
Ávila	BD5	R. Hernández C. B. (Muñogalindo)
	BD6	La Garrosa (Solosancho)
	BD7	La Candelaria (Sotalbo)
	BD8	I. López Hernández (La Horcajada)
Zamora	BD9	Repostería Mateos S.A. (Entrala)
	BD10	Mayagus S.L. (Mayalde)
	BD11	A. Rodríguez (Videmala)
	BD12	Sta. María de la Vega (Bretó de la Ribera)
	BD13	Lomar (Ayoó de Vidriales)
	BD14	Santa Marina (Aguilar de Tera)
	BD15	Montero Mezquita (Gallegos del Río)
León	BD16	Pedro González (Val de San Lorenzo)

**Table 3:** Cereal grains and flours used for the isolation of yeasts.

Sample	Grain	Source
EE1	5 cereal mixture	Emilio Esteban (Valladolid, Spain)
EE4	rye	Emilio Esteban (Valladolid, Spain)
EE5	barley	Emilio Esteban (Valladolid, Spain)
EE6	wheat	Emilio Esteban (Valladolid, Spain)
EE9	oat	Emilio Esteban (Valladolid, Spain)
T1	tritordeum—aucan	* Irnasa (CSIC) (Salamanca, Spain)
T4	tritordeum—aucan	(Jaén, Spain)
T6	tritordeum—aucan	(Córdoba, Spain)
T7	tritordeum—bulel	(Córdoba, Spain)
Gtit	tritordeum	(Bari, Italy)
	**Flour**	
HS2	WM rye	Stone, Rincón del Segura (Albacete, Spain)
HS1	WM wheat	Stone, Rincón del Segura (Albacete, Spain)
H1	wheat W180–200	Cylinders, Molinos del Duero (Zamora, Spain)
H2	WM wheat	Cylinders, La Vilafranquina (Ávila, Spain)
H3	wheat W130–150	Cylinders, Molinos del Duero (Zamora, Spain)
H4	wheat “Oromas”	Cylinders, La Vilafranquina (Ávila, Spain)
H5	T. Zamorana W230–250	Stone, Molinos del Duero (Zamora, Spain)
H6	WM tritordeum	(Málaga, Spain)
H7	tritordeum	(Málaga, Spain)
T9	tritordeum—aucan	(Barcelona, Spain)
ACoF	tritordeum—aucan	(Córdoba, Spain)
AJF	tritordeum—aucan	(Jaén, Spain)
BCF	tritordeum—bulel	(Sevilla, Spain)
BCoF	tritordeum—bulel	(Córdoba, Spain)
BJF	tritordeum—bulel	(Jaén, Spain)
ACoI	WM tritordeum—aucan	(Córdoba, Spain)
BCI	WM tritordeum—bulel	(Sevilla, Spain)
BCoI	WM tritordeum—bulel	(Córdoba, Spain)
THI	WM tritordeum—aucan	(Sevilla, Spain)
HRTit	tritordeum—bulel	(Bari, Italy)

The cereal grains and flours, the providers or milling companies, and the provinces in Spain are indicated, as well as the milling procedure when known (stone or cylinders). The tritordeum grains of the varieties aucan and bulel, the corresponding flours, and the novel and experimental crop varieties cultured at several geographical locations were provided by Agrasys S.L. (Barcelona, Spain). * Grains from a new experimental variety of tritordeum provided by the IRNASA Institute (Spanish Research Council, CSIC; Salamanca, Spain). The wholemeal flours are indicated as WM and the others were all refined flours.

**Table 4:** Identification of representative yeast strains by ITS types and RAPD groups.

Representative Yeast Strains (Laboratory Code)	ITS Type	RAPD Groups	Closest Type Strain Accession Numbers 5.8-ITS/LSU (D1/D2)	ITS/LSU (D1/D2) Similarity (%)
P7FP8, P6FP8, P5FP3, P4FP5, P5FP8, P5FP10, P6FP2, P1FP9, P3FP5, P1FP2	I	A, B, C, D, E, F, G, I, J, K	*Kazachstania bulderi* CBS 8638^T^ KY103628/KY107908	100/100
MTB-1	I	H	*Kazachstania humilis* CBS 5658^T^ KY102142/KY106507	97.7/100
H41	I	L	*Kazachstania servazzii* CBS 4311^T^ KY103668/KM454442	99.9/99.8
P2A5	I	M	*K. servazzii* CBS 4311^T^ KY103668/KM454442	99.6/99.8
P4A6, GTi7	II	A, C	*Meyerozyma guilliermondii* CBS 2030^T^ NR_111247/KY108542	100/99.8
Ag2	II	D	*Meyerozyma carpophila* CBS 5256^T^ MK394110/KY106386	100/99.8
H6.3	II	B	*M. carpophila* CBS 5256^T^ MK394110/KY106386	99.8/99.8
ME2FP5, HRTi8, HRTi7	III	A, H, J	*Pichia fermentans* CBS 187^T^ KY104545/KY108804	98.4/99.5
EE9B	III	L	*P. fermentans* CBS 187^T^ KY104545/KY108804	99.1/99.5
ME2FP2, ME1A5, ME3A1	III	F, G, I	*P. fermentans* CBS 187^T^ KY104545/KY108804	99.8/99.5
H5.2, P6FP4, ME2A1, ME4A7, P3FP2, EE1A	III	B, C, D, E, K, M	*P. fermentans* CBS 187^T^ KY104545/KY108804	100/99.5
YMAS12, ME1A8	IV	A, B	*Saccharomyces cerevisiae* CBS 1171^T^ AB018043/KC881066	100/100
GTi5	IV	D	*S. cerevisiae* CBS 1171^T^ AB018043/KC881066	99.6/100
MJA2.1, ME5FP10	IV	E, G	*S. cerevisiae* CBS 1171^T^ AB018043/KC881066	99.8/100
YMAS3, YMAS2	IV	C, F	*S. cerevisiae* CBS 1171^T^ AB018043/KC881066	99.9/100
ME3FP9, ME5A5, P3D6, P6A10, P7F10, P5A7, P5A8, P5A2, ME6FP10, ME3A7, ME3A2, H.S.1.1, ME2A2	V	A, B, C, D, E, F, G, H, I, L, M, O, Q	*Torulaspora delbrueckii* CBS 1146^T^ KY105617/AJ508558	100/100
ME2FP10, ME3FP3, ME2FP7, ME4FP1, ME5FP6	V	J, K, N, R	*T. delbrueckii* CBS 1146^T^ KY105617/AJ508558	99.9/100
T9, GTi1, ME2FP3, ME1FP9	VI	A, C, D, E	*Wickerhamomyces anomalus* CBS 5759^T^ KY105894/EU057562	100/100
ME5FP8	VI	B	*W. anomalus* CBS 5759^T^ KY105894/EU057562	98.7/100

**Table 5:** Comparative analysis of yeast isolation versus estimated relative abundance in selected MDs.

MDs:	MD7 Wheat, Firm				MD8 Wheat, Liquid				MD11 Tritordeum, Firm				MD12 Tritordeum, Liquid			
Fermentation step:	**BS1 FP**				**BS1 FP**				**BS1 FP**				**BS1 FP**			
	**I**	**%**	**I**	**%**	**I**	**%**	**I**	**%**	**I**	**%**	**I**	**%**	**I**	**%**	**I**	**%**
*Kazachstania bulderi*	-	-	-	-	-	-	4	56	-	-	-	-	-	-	9	21.5
*Kazachstania servazzii*	1	12	-	5	10	43	-	8	1	46	-	31.5	-	40	-	20
*Pichia fermentans*	3	40	4	7.5	-	27.5	6	35	-	4	-	1.5	-	33	1	48
*Saccharomyces cerevisiae*	-	5	-	-	-	0.7	-	0.1	1	1.6	-	15.7	-	0.2	-	0.1
*Torulaspora delbrueckii*	4	37	3	33	-	26	-	0.1	5	48	10	25	10	12	-	0.3
*Wickerhamomyces anomalus*	-	2	3	54	-	1	-	0.7	-	0.1	-	23	-	0.8	-	8

I: Number of yeast strains isolated in culture. %: Yeast species abundance in the fungal microbiome. When a species was identified in a sample by the two different methods, a green color was used. When a species abundance is higher than 10% and strains were not culture-isolated, a yellow color is used.

**Table 6:** Dough leavening and maximal volume for 17 S*accharomyces cerevisiae* strains.

	Fermentation Rate (mL/min)		Maximal Volume, mL (min)	
Strain	Wheat	Tritordeum	Wheat	Tritordeum
Ay2	331 ± 4	393 ± 4	14.1 ± 1.3 (75′)	16.4 ± 0.2 (60′)
Ent1	352 ± 4	444 ± 3	14.9 ± 1.3 (75′)	18.7 ± 0.5 (60′)
SFG1	321 ± 1	479 ± 1	14.3 ± 0.4 (60′)	20.2 ± 3.1 (60′)
SFG3	324 ± 1	416 ± 1	14.4 ± 0.5 (60′)	19.9 ± 0.7 (75′)
MJA2.1	394 ± 2	343 ± 2	16.0 ± 0.0 (75′)	15.8 ± 1.1 (75′)
ME5FP10	314 ± 3	428 ± 1	13.2 ± 1.8 (60′)	19.3 ± 0.4 (60′)
ME7FP6	373 ± 5	442 ± 1	13.3 ± 1.7 (60′)	20.4 ± 0.4 (60′)
Ag2	25 ± 0	73 ± 5	1.3 ± 0.4 (90′)	3.84 ± 0.2 (90′)
H.S.1.1	153 ± 19	174 ± 18	9.3 ± 0.7 (90′)	13.4 ± 0.2 (90′)
YMAS2	292 ± 3	452 ± 2	12.6 ± 1.6 (60′)	21.0 ± 2.0 (60′)
YMAS5	211 ± 3	327 ± 1	10.3 ± 1.3 (75′)	16.3 ± 0.7 (75′)
YMAS12	337 ± 6	443 ± 3	14.8 ± 0.2 (60′)	20.6 ± 2.2 (60′)
YMAS23	294 ± 5	431 ± 2	13.6 ± 2.0 (60′)	19.8 ± 1.4 (60′)
YMAS36	329 ± 14	488 ± 6	11.8 ± 1.4 (60′)	19.3 ± 0.7 (60′)
YMAS44	314 ± 1	381 ± 2	14.5 ± 0.7 (75′)	18.1 ± 0.4 (60′)
YMAS60	403 ± 30	623 ± 54	15.0 ± 0.7 (45′)	26.1 ± 3.4 (60′)
CS	343 ± 1	426 ± 2	14.2 ± 2.0 (60′)	20.0 ± 0.8 (60′)

Fermentation parameters for the 17 *Saccharomyces cerevisiae* strains in Figure 4. Fermentation rates in wheat and tritordeum estimated at the lineal phase of volume increase in graduated tubes are given in μL/min. The maximal volumes of dough leavening reached at 60–90 min, depending on the strain, are given in mL/min. CS: Commercial Strain (ABM-CL). Values are the mean of three independent experiments and the standard deviations are shown. Strain origin is shown in Table S1.

**Table 7:** CO_2_ production rate and maximal volume in flour-free MLD for 17 yeast strains.

	CO_2_ Production Rate(cm^3^/h)	Volume of CO_2_ (cm^3^)		
Strain		10 h	12 h	18 h
YMAS2	44 ± 3.4	329 ± 21	335 ± 35	343 ± 40
YMAS5	36 ± 2.9	405 ± 16	423 ± 14	565 ± 25
YMAS12	43 ± 3.4	388 ± 50	402 ± 30	466 ± 94
YMAS23	37 ± 2.4	322 ± 15	339 ± 9	376 ± 19
YMAS36	29 ± 0.5	309 ± 9	329 ± 38	400 ± 13
YMAS44	37 ± 2.2	374 ± 52	390 ± 28	471 ± 12
YMAS60	31 ± 0.3	327 ± 14	337 ± 1	411 ± 90
Ay2	42.± 0.4	354 ± 20	363 ± 6	379 ± 16
Ent1	42 ± 3.0	348 ± 16	358 ± 1	369 ± 1
SFG1	44 ± 1.5	353 ± 35	367 ± 15	385 ± 19
SFG3	42 ± 2.3	331 ± 49	341 ± 35	361 ± 51
MJA2.1	43 ± 1.0	340 ± 16	354 ± 4	375 ± 0
ME5FP10	39 ± 1.3	302 ± 66	323 ± 37	380 ± 1
ME7FP6	42 ± 0.5	319 ± 61	342 ± 29	397 ± 6.2
Ag2	12 ± 0.8	93 ± 17	100 ± 23	118 ± 49
H.S.1.1	15 ± 0.2	101 ± 31	119 ± 49	173 ± 11
CS	39 ± 3.1	305± 16	345 ± 6	403 ± 12

The gas production rates (cm^3^ CO_2_/h) were estimated for each yeast strain at the lineal phase of volume increase (~2 to 5 h). Volumes of CO_2_ recorded by the Ankom apparatus at 10, 12, and 18 h of fermentation are given. Values are the mean of three independent experiments and the standard deviations are shown.

**Table 8:** Maltase activity for 12 *Saccharomyces cerevisiae* strains.

	(U) nM pNP/min/mg Protein	
Strain	YPD	YPM
YMAS2	108 ± 4.5	4897 ± 444
YMAS5	50 ± 2.0	4127 ± 134
YMAS12	98 ± 3.1	3475 ± 344
YMAS23	51 ± 7.1	5636 ± 319
YMAS44	77 ± 2.7	4082 ± 250
Ay2	88 ± 2.0	5092 ± 173
Ent1	252 ± 2.9	3773 ± 330
SFG1	80 ± 0.8	5475 ± 200
SFG3	71 ± 10	4613 ± 314
MJA2.1	11 ± 6.7	3513 ± 143
ME5FP10	61 ± 6.8	4149 ± 203
ME7FP6	97 ± 0.2	3099 ± 101
CS	103 ± 6.5	4540 ± 265

Maltase activity (alpha-1,4-glucosidase) quantified in the WCE of cells grown to an A600 of ~1.0 under repressing (YPD, 2% dextrose) or derepressing conditions (YPM, 2% maltose) of maltase production. Enzymatic units (U) are given as nanomoles of para-nitrophenol (pNP) released from para-nitrophenyl-alpha-1,4-glucopyranoside (pNPG) per minute per mg of total protein (WCE). CS: commercial strain used for lean doughs (ABM-CL). Values are the mean ± SD values in two independent biological replicates.

**Table 9:** Invertase activity for 37 yeast strains.

	(U) mM Glucose /min/mg WCE	
Strain	YPD	YPS
YMAS1	1412	5618
YMAS2	5109	9383
YMAS3	3061	7738
YMAS 5	5908	9300
YMAS5	3110	7259
YMAS7	1504	4512
YMAS8	2802	11,567
YMAS12	3711	7671
YMAS23	4211	14,184
YMAS 35	357	3602
YMAS 36	2404	6832
YMAS 44	2758	6147
YMAS 47	2512	3464
YMAS 52	348	3997
YMAS 59	3862	9392
YMAS 60	3546	9111
Ay2	4374	8595
Ag4	1864	2088
Ag7	2232	4808
Ent1	5129	13,453
Ent3	3663	7222
Ent5	2198	4519
Br1	2854	4235
Br7	4295	8404
Gal1	1283	6667
SFG1	4327	6104
SFG2	3506	5058
SFG3	5164	7153
ME7FP6	521	4660
Pc1	4277	7873
Pc2	12,886	13,646
MFa6	4448	8079
MFa7	4018	6131
MFb3	2721	5447
MFb5	4000	6029
Bc4	3950	12,541
Bc5	4719	8231
Ag2	1027	1524
H.S.1.1	786	817
CD	1029	4236

Invertase activity (beta-fructo-furanosidase) was quantified in the WCE of cells grown to an A_600_ of ~1.0 in YPD medium (2% dextrose) for repressing conditions or in YPS (2% sucrose) for derepressing conditions of invertase production. Values obtained in a representative experiment are shown. Enzymatic units (U) given as millimoles of glucose liberated from sucrose per minute per mg of total protein (WCE). CD: Baker’s yeast for sweet doughs.

**Table 10:** Vitamin requirements of 433 yeast isolates.

Yeast Species	Number of Isolates	Thiamine (B_1_)	Nicotinic Acid (B_3)_	Biotin (B_7_)	
*Kazachstania bulderi*	33 (MD)	−	−	+	
*K. bulderi*	2 (MD)	+	−	−	
*K. bulderi*	1 (MD)	−	−	−/+	
*Kazachstania humilis*	7 (MD)	−	−	+	
*Kazachstania servazzii*	22 (21 MD, 1F)	+	+	+	
*Meyerozyma carpophila*	2 (BD)	−	−	+	
*M. carpophila*	2 (F)	+	−	−	
*Meyerozyma guilliermondii*	8 (MD)	−	−	−/+	
*M. guilliermondii*	2 (G)	−	−	+	
*Pichia fermentans*	47 (MD)	+	−	−	
*P. fermentans*	3 (MD)	−	−	+	
*P. fermentans*	3 (MD)	+	+	+	
*P. fermentans*	9 (F)	+	−	−	
*Saccharomyces cerevisiae*	36 (MD)	−	−	+	
*S. cerevisiae*	121 (BD)	−	−	+	
*S. cerevisiae*	1 (G)	+	−	−	
*Torulaspora delbrueckii*	73 (71 MD, 2 F)	−	−	+	
*T. delbrueckii*	37 (35 MD, 2 F)	−	−	−/+	
*T. delbrueckii*	4 (3 MD, 1 F)	−	−	−	
*Wickerhamomyces anomalus*	19 (8 MD, 7 H, 4 G)	−	−	−	

In parentheses, the source of origin: MD, mother dough; BD, bakery dough; F, Flour; G, Grain. −, not required; +, required; −/+2 days of growth delay observed in cultures without biotin.

**Table 11:** Riboflavin production by the selected strains of *Meyerozyma* spp.

Species and Strain Codes(Matrix) *	Riboflavin Concentration ** (µg/mL Medium)		
	Iron Concentration (Fe^+++^)		
	1.23 µM	12.3 µM	61.5 µM
*Meyerozyma carpophila* H6.1 (F)	4.15 ± 0.13	4.30 ± 0.19	4.40 ± 0.17
*M. carpophila* YMAS59 (BD)	4.20 ± 0.10	4.40 ± 0.30	4.30 ± 0.20
*M. carpophila* Ag2 (BD)	4.10 ± 0.11	4.00 ± 0.12	4.10 ± 0.30
*Meyerozyma guilliermondii* GTi7 (F)	14.82 ± 0.10	3.80 ± 0.21	0.40 ± 0.01
*M. guilliermondii* P4A6 (MD)	16.10 ± 0.31	4.20 ± 0.51	0.50 ± 0.00
*M. guilliermondii* P4A9 (MD)	16.08 ± 0.42	4.20 ± 0.34	0.50 ± 0.01

* (F), Flour; (BD), Baking Dough (MD), Mother Dough; (G) grain. ** mean ± SD values in three independent biological replicates.

**Table 12:** Extracellular hydrolytic activities of 23 selected yeast strains.

Species and Strain Codes (Source) *	Bioassays (halo in mm **)				Activities by Growth ***	
	Esterase	Protease	Glyadinase	Pectinase	Phytase	Cellobiase
*Kazachstania bulderi* P1FP9 (MD)	4	0	0	0	−	−
*K. bulderi* P3FP5 (MD)	4	0	0	0	−	−
*Kazachstania humilis* MTB−1 (MD)	6	0	0	0	+	−
*K. humilis* MBE−4 (MD)	6	0	0	0	+	−
*Kazachstania servazzii* H4.1 (F)	5	6	0	0	−	−
*K. servazzii* P2A5 (MD)	5	6	0	0	−	−
*Meyerozyma carpophila* H6.3 (F)	2	5	0	0	−	+
*M. carpophila* Ag2 (BD)	2	5	0	0	−	+
*Meyerozyma guilliermondii* GTi7 (F)	2	4	0	0	−	+
*M. guilliermondii* P4A6 (MD)	2	4	0	0	−	+
*M. guilliermondii* P4A9 (MD)	2	4	0	0		
*Pichia fermentans* EE1A (F)	0	15	4	0	−	+
*P. fermentans* HRTi7 (F)	0	15	4	0	−	+
*P. fermentans* ME2FP5 (MD)	0	15	4	0	−	+
*P. fermentans* P6FP4 (MD)	0	15	4	0	−	+
*Torulaspora delbrueckii* H1.2 (F)	5	0	0	0	−	+
*T. delbrueckii* ME2FP7 (MD)	5	0	0	0	−	+
*T. delbrueckii* ME6FP10 (MD)	5	0	0	0	−	+
*T. delbrueckii* P6A10 (MD)	5	0	0	0	−	+
*Wickerhamomyces anomalus* T9 (G)	12	21	4	4	+	+
*W. anomalus* HRTi1 (F)	12	21	4	4	+	+
*W. anomalus* ME1FP9 (MD)	12	21	4	4	+	+
W. *anomalus* YMAT1 (MD)	12	21	4	4	+	+

* (MD), Mother Dough; (BD), Bakery Dough; (F), Flour; (G), Grain. ** Measured from the outer edge of the colony to the outer limit of the halo. *** + with and − without enzymatic activity (Section 2.4.6).

## Data Availability

Data is available upon request to corresponding author.

## Supplementary Materials

The following are available online at https://www.mdpi.com/2076-2607/9/1/47/s1. Figure S1: Different sizes obtained for the amplification of the 5.8S-ITS region in the strains from six yeast genera. *Torulaspora* (lane 1, type I), *Meyerozyma* (lane 2, type II), *Kazachstania* (lane 3, type III), *Pichia* (lane 4, type IV), *Wickerhamomyces* (lane 5, type V), and *Saccharomyces* (lane 6, type VI), Figures S2 to S7: Dendrograms obtained for the strains from ITS type I to VI, using Jaccard’s coefficient and UPGMA analysis of the RAPD profiles, Table S1: Summary of the 433 presumptive wild yeasts isolated from fermented doughs and raw cereal matrices and the corresponding species, Table S2: Results of the identification of yeast species in the 433 isolates from this study, Table S3: Species and number of yeasts isolated from MDs relative to the flour, consistency and fermentation time, Table S4: The 81 discarded isolates and matrices of origin.
