# Supplementary material for: Yeast Biodiversity in Fermented Doughs and Raw Cereal Matrices and the Study of Technological Traits of Selected Strains Isolated in Spain
Source: Microorganisms. 2020 Dec 26;9(1):47. doi: 10.3390/microorganisms9010047 (PMC7824024; doi:10.3390/microorganisms9010047)
Supplement: Supplementary file 1 [file microorganisms-09-00047-s001.zip › microorganisms-1035953-supplementary-revised/microorganisms-1035953-supplementary-revised.docx]

**APPENDIX A**

**SUPPLEMENTARY FIGURES AND TABLES**

**FIGURE S1**

**
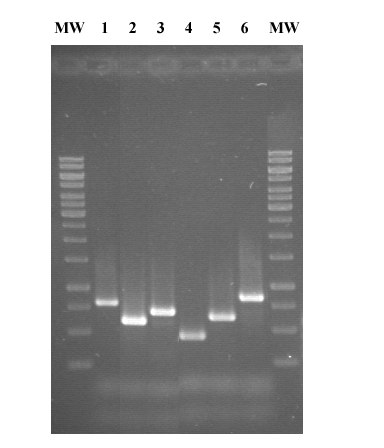
**

**Figure S1.** Different sizes obtained for the amplification of the 5.8S-ITS region in the strains from six yeast genera. *Torulaspora* (lane 1, type I), *Meyerozyma* (lane 2, type II), *Kazachstania* (lane 3, type III), *Pichia* (lane 4, type IV), *Wickerhamomyces* (lane 5, type V), and *Saccharomyces* (lane 6, type VI).

**FIGURE S2**

**Figure S2.** Dendrogram obtained for the *Torulaspora* strains from ITS type I using Jaccard’s coefficient and UPGMA analysis of the RAPD profiles.

**FIGURE S3**

Figure S3. Dendrogram obtained for the *Meyerozyma* strains from ITS type II using Jaccard’s coefficient and UPGMA analysis of the RAPD profiles.

**FIGURE S4**

**Figure S4.** Dendrogram obtained for the *Kazachstania* strains from ITS type III using Jaccard’s coefficient and UPGMA analysis of the RAPD profiles.

**FIGURE S5**

**Figure S5.** Dendrogram obtained for the *Pichia* strains from ITS type IV using Jaccard’s coefficient and UPGMA analysis of the RAPD profiles.

**FIGURE S6**

**Figure S6.** Dendrogram obtained for the *Wickerhamomyces* strains from ITS type V using Jaccard’s coefficient and UPGMA analysis of the RAPD profiles

FIGURE S7

**Figure S7.** Dendrogram obtained for the *Saccharomyces* strains from ITS type VI using Jaccard’s coefficient and UPGMA analysis of the RAPD profiles

**Table S1**. Summary of the 433 presumptive wild yeasts isolated from fermented doughs and raw cereal matrices and the corresponding species.

| **(1)** **MOTHER DOUGHS** | | |
| --- | --- | --- |
| **MD1-M14 (this work)** | **Cereal Flour** |  |
| **ISOLATE CODE** | **Back slopping step** | ***Yeast species*** |
| MD1- firm | wheat (W180–200) |  |
| ME1A2, ME1A5, ME1A7 | BS1 | *Pichia fermentans* |
| ME1A8, | BS1 | *Saccharomyces cerevisiae* |
| ME1A1, ME1A3, ME1A4, ME1A6 | BS1 | *Torulaspora delbrueckii* |
| ME1FP1, ME1FP3, ME1FP4, ME1FP5, ME1FP6, ME1FP7, ME1FP8 | FP | *Torulaspora delbrueckii* |
| ME1FP2 | FP | *Saccharomyces cerevisiae* |
| ME1FP9, ME1FP10 | FP | *Wickerhamomyces anomalus* |
| MD2- liquid | wheat (W180–200) |  |
| P1J1 | BS1 | *Torulaspora delbrueckii* |
| P1J2, P1J3, P1J5, P1J6, P1J7, P1J8 | BS1 | *Saccharomyces cerevisiae* |
| P1J4, P1J9 | BS1 | *Pichia fermentans* |
| P1J10 | BS1 | *Kazachstania servazzii* |
| P1FP1, P1FP4 | FP | *Saccharomyces cerevisiae* |
| P1FP2, P1FP6, P1FP7, P1FP8, P1FP9, P1FP10 | FP | *Kazachstania bulderi* |
| P1FP3, P1FP5 | FP | *Pichia fermentans* |
| MD3-firm | wheat (W13–150) |  |
| ME3A5, ME3A6 | BS1 | *Kazachstania servazzii* |
| ME3A1, ME3A4 | BS1 | *Pichia fermentans* |
| ME3A8 | BS1 | *Saccharomyces cerevisiae* |
| ME3A2, ME3A3, ME3A7 | BS1 | *Torulaspora delbrueckii* |
| ME3FP1, ME3FP3, ME3FP4, ME3FP5, ME3FP6, ME3FP7, ME3FP8, ME3FP9 | FP | *Torulaspora delbrueckii* |
| ME3FP10, ME3FP2 | FP | *Saccharomyces cerevisiae* |
| MD4-liquid | wheat (W130–150) |  |
| P3D1, P3D10 | BS1 | *Saccharomyces cerevisiae* |
| P3D2, P3D3, P3D4, P3D5, P3D6, P3D7, P3D8, P3D9 | BS1 | *Torulaspora delbrueckii* |
| P3FP1 | FP | *Saccharomyces cerevisiae* |
| P3FP2, P3FP3, P3FP4, P3FP7, P3FP9 | FP | *Pichia fermentans* |
| P3FP5, P3FP6, P3FP8, P3FP10 | FP | *Kazachstania bulderi* |
| MD5-firm | Tradicional Zamorana  W230-250 (TZM) |  |
| ME5A1 | BS1 | *Pichia fermentans* |
| ME5A2, ME5A4, ME5A5, ME5A7, ME5A8 | BS1 | *Torulaspora delbrueckii* |
| ME5A3, ME5A6 | BS1 | *Kazachstania servazzii* |
| ME5FP1, ME5FP2, ME5FP3, ME5FP4, ME5FP5, ME5FP6, ME5FP7 | FP | *Torulaspora delbrueckii* |
| ME5FP8 | FP | *Wickerhamomyces anomalus* |
| ME5FP9, ME5FP10 | FP | *Saccharomyces cerevisiae* |
| MD6-liquid | Tradicional Zamorana  W230–250 (TZM) |  |
| P4A1, P4A4 | BS1 | *Kazachstania servazzii* |
| P4A2, P4A3, P4A5, P4A6, P4A7, P4A8, P4A9, P4A10 | FP | *Meyerozyma guilliermondii* |
| P4FP1, P4FP2, P4FP4, P4FP7, P4FP9, P4FP10 | FP | *Pichia fermentans* |
| P4FP3, P4FP5, P4FP6, P4FP8 | FP | *Kazachstania bulderi* |
| MD7-firm | Whole meal wheat (WMW) |  |
| ME2A1, ME2A4 | BS1 | *Pichia fermentans* |
| ME2A2, ME2A3, ME2A6, ME2A7, ME2A8 | BS1 | *Torulaspora delbrueckii* |
| ME2A5 | BS1 | *Kazachstania servazzii* |
| ME2FP1, ME2FP3, ME2FP8 | FP | *Wickerhamomyces anomalus* |
| ME2FP2, ME2FP5, ME2FP6, ME2FP9 | FP | *Pichia fermentans* |
| ME2FP4, ME2FP7, ME2FP10 | FP | *Torulaspora delbrueckii* |
| MD8-liquid | Whole meal wheat (WMW) |  |
| P2A1, P2A2, P2A3, P2A4, P2A5, P2A6, P2A7, P2A8, P2A9, P2A10 | BS1 | *Kazachstania servazzii* |
| P2FP1, P2FP3, P2FP4, P2FP6, P2FP7, P2FP8 | FP | *Pichia fermentans* |
| P2FP2, P2FP5, P2FP9 | FP | *Kazachstania bulderi* |
| MD9-firm | tritordeum W100–110 (Tr) |  |
| ME7A1, ME7A3, ME7A4, ME7A5, ME7A6, ME7A7 | BS1 | *Torulaspora delbrueckii* |
| ME7A2 | BS1 | *Saccharomyces cerevisiae* |
| ME7A8 | BS1 | *Kazachstania servazzii* |
| ME7FP1, ME7FP2, ME7FP3, ME7FP4, ME7FP5, ME7FP6, ME7FP7, ME7FP8, ME7FP9, ME7FP10 | FP | *Saccharomyces cerevisiae* |
| MD10-liquid | tritordeum W100–110 (Tr) |  |
| P6A1, P6A2, P6A3, P6A4, P6A5, P6A6, P6A7, P6A8, P6A9, P6A10 | BS1 | *Torulaspora delbrueckii* |
| P6FP1, P6FP3, P6FP4, P6FP5, P6FP6, P6FP10 | FP | *Pichia fermentans* |
| P6FP2, P6FP7, P6FP8, P6FP9 | FP | *Kazachstania bulderi* |
| MD11-firm | Whole meal tritordeum (WMtr) |  |
| ME6A1, ME6A4, ME6A5, ME6A6, ME6A7 | BS1 | *Torulaspora delbrueckii* |
| ME6A2 | BS1 | *Saccharomyces cerevisiae* |
| ME6A3 | BS1 | *Kazachstania servazzii* |
| ME6A8 | BS1 | *Pichia fermentans* |
| ME6FP1, ME6FP2, ME6FP3, ME6FP4, ME6FP5, ME6FP6, ME6FP7, ME6FP8, ME6FP9, ME6FP10 | FP | *Torulaspora delbrueckii* |
| MD12-liquid | Whole meal tritordeum (WMtr) |  |
| P5A1, P5A2, P5A3, P5A4, P5A5, P5A6, P5A7, P5A8, P5A9, P5A10 | BS1 | *Torulaspora delbrueckii* |
| P5FP1, P5FP2, P5FP3, P5FP4, P5FP5, P5FP6, P5FP7, P5FP8, P5FP10 | FP | *Kazachstania bulderi* |
| P5FP9 | FP | *Pichia fermentans* |
| MD13-firm | wheat (Oromas) |  |
| ME4A1, ME4A3, ME4A5, ME4A7 | BS1 | *Pichia fermentans* |
| ME4A2, ME4A6 | BS1 | *Torulaspora delbrueckii* |
| ME4A4 | BS1 | *Kazachstania servazzii* |
| ME4FP1, ME4FP3, ME4FP4, ME4FP5, ME4FP6, ME4FP7, ME4FP8, ME4FP9 | FP | *Torulaspora delbrueckii* |
| ME4FP2 | FP | *Saccharomyces cerevisiae* |
| ME4FP10 | FP | *Pichia fermentans* |
| MD14-liquid | Mix of the 6 previous flours (MO6F) |  |
| P7F1, P7F2, P7F4 | BS1 | *Saccharomyces cerevisiae* |
| P7F3, P7F5, P7F6, P7F7, P7F8, P7F9, P7F10 | BS1 | *Torulaspora delbrueckii* |
| P7FP1, P7FP2, P7FP3, P7FP8, P7FP9, P7FP10 | FP | *Kazachstania bulderi* |
| P7FP4, P7FP5, P7FP6, P7FP7 | FP | *Pichia fermentans* |
|  |  |  |
| **MD15-MD21 (other origin)** | **Flour and MD type** | ***Yeast species*** |
| MD15-Firm ^a^ | wheat |  |
| MJA2.1, MJA2.2 | Homemade with fruits & yogurt | *Saccharomyces cerevisiae* |
| MD16-Firm ^a^ | tritordeum (W100–110) |  |
| YMAT1, YMAT2 | Homemade, Type I | *Wickerhamomyces anomalus* |
| MD17- Firm ^a^ | Whole meal tritordeum |  |
| YMATi2, YMATi3, YMATi4 | Homemade, Type I | *Pichia fermentans* |
| YMATi5, YMATi1 | Homemade, Type I | *Saccharomyces cerevisiae* |
| MD18-Firm ^b^ | tritordeum (W100–110) |  |
| Bc4, Bc5, Bc6, Bc7 | Type I | *Saccharomyces cerevisiae* |
| MTB-1, MTB-2, MTB-3 | Type I | *Kazachstania humilis* |
| MD19-Firm ^b^ | wheat “T80” (W200–220) |  |
| MBE-1, MBE-2, MBE-3, MBE-4 | Type I, | *Kazachstania humilis* |
| MD20-Firm ^c^ | wheat |  |
| MFa1, MFa2, MFa3, MFa4, MFa5, MFa6, MFa7 | Travelling Baker (TB) | *Saccharomyces cerevisiae* |
| MD21-Firm ^c^ | wheat |  |
| MFb1, MFb2, MFb3, MFb4, MFb5, MFb6, MFb7, MFb8 | Fred Bakeries (FB) | *Saccharomyces cerevisiae* |
| **(2) BAKERY DOUGHS** | | |
| **STRAIN CODE** | **(Town, Province)** | ***Yeast Species*** |
| YMAS1, YMAS2, YMAS3, YMAS4, YMAS5, YMAS6, YMAS7, YMAS8, YMAS9, YMAS10, YMAS11, YMAS12, YMAS13 | BD1 (Cabrerizos, Salamanca) | *Saccharomyces cerevisiae* |
| YMAS45, YMAS46, YMAS47, YMAS48, YMAS49, YMAS52 | BD2 (San Morales, Salamanca) | *Saccharomyces cerevisiae* |
| SFG1, SFG2, SFG3, SFG9, SFG10 | BD3 (San Felices  de los Gallegos, Salamanca) | *Saccharomyces cerevisiae* |
| YMAS120 | BD4 (Lumbrales, Salamanca) | *Saccharomyces cerevisiae* |
| YMAS14, YMAS15, YMAS21, YMAS22 | BD5 (Muñogalindo, Ávila) | *Saccharomyces cerevisiae* |
| YMAS18, YMAS19, YMAS20 | BD6 (Solosancho, Ávila) | *Saccharomyces cerevisiae* |
| YMAS16, YMAS23, YMAS24 | BD7 (Sotalbo, Ávila) | *Saccharomyces cerevisiae* |
| YMAS59 | BD8 (La Horcajada, Ávila) | *Meyerozyma carpophila* |
| YMAS55, YMAS57, YMAS60, YMAS61, YMAS62, YMAS63 | BD8 (La Horcajada, Ávila) | *Saccharomyces cerevisiae* |
| YMAS27, YMAS28, YMAS29, YMAS31, YMAS32 YMAS34, YMAS35, YMAS36, YMAS38 | BD9 (Entrala, Zamora) | *Saccharomyces cerevisiae* |
| Ent1, Ent2, Ent3, Ent4, Ent5, Ent6, Ent7 | BD9 (Entrala, Zamora) | *Saccharomyces cerevisiae* |
| YMAS39, YMAS41, YMAS42, YMAS43, YMAS44 | BD10 (Mayalde, Zamora) | *Saccharomyces cerevisiae* |
| Vid1, Vid2, Vid3, Vid4, Vid5 | BD11 (Videmala, Zamora) | *Saccharomyces cerevisiae* |
| Br1, Br2, Br3, Br4, Br5, Br 6, Br7, Br8, Br9, Br10 | BD12 (Bretó de la Ribera, Zamora) | *Saccharomyces cerevisiae* |
| Ay1, Ay2, Ay3, Ay4, Ay5, Ay6 | BD13 (Ayoó de Vidriales, Zamora) | *Saccharomyces cerevisiae* |
| Ag1, Ag3, Ag4, Ag5, Ag6, Ag7 | BD14 (Aguilar de Tera, Zamora) | *Saccharomyces cerevisiae* |
| Ag2 | BD14 (Aguilar de Tera, Zamora) | *Meyerozyma carpophila* |
| Gal1, Gal2, Gal3, Gal4, Gal5, Gal6 | BD15 (Gallegos del Río, Zamora) | *Saccharomyces cerevisiae* |
| LEV53, LEV54, LEV55, LEV56, LEV57, LEV58 | BD16 (Val de San Lorenzo, León) | *Saccharomyces cerevisiae* |
| **(3) RAW MATRICES** | | |
| **STRAIN CODE** | **Cereal and Matrix** | ***Yeast species*** |
|  | Grain |  |
| EE5A | barley | *Pichia fermentans* |
| EE6A, EE6C | wheat | *Wickerhamomyces anomalus* |
| EE9B | oat | *Pichia fermentans* |
| EE1A | 5 cereal grains mixture | *Pichia fermentans* |
| GTi1, GTi4 | tritordeum (Italy) | *Wickerhamomyces anomalus* |
| GTi5 | tritordeum (Italy) | *Saccharomyces cerevisiae* |
| GTi7, GTi9 | tritordeum (Italy) | *Meyerozyma guilliermondii* |
|  | Flour |  |
| H1.2 | wheat, W180–200 | *Torulaspora delbrueckii* |
| H3.2, H3.3 | wheat, W130–150 | *Torulaspora delbrueckii* |
| H4.1 | wheat, Oromas | *Kazachstania servazzii* |
| H5.1, H5.2 | Tradicional Zamorana  W230–250 (TZM) | *Pichia fermentans* |
| H2.1 | Whole meal wheat (WMW) | *Pichia fermentans* |
| H2.2 | Whole meal wheat (WMW) | *Torulaspora delbrueckii* |
| T9, T11, T12, T13 | tritordeum W100–110 (tr) | *Wickerhamomyces anomalus* |
| H6.1, H6.3 | Whole meal tritordeum (WMtr) | *Meyerozyma carpophila* |
| HRTi7, HRTi8 | tritordeum (Bari, Italy) | *Pichia fermentans* |
| HRITi1, HRITi3, HRITi6 | tritordeum (Bari, Italy) | *Wickerhamomyces anomalus* |
| H.S.1.1 | Whole meal wheat  (Albacete, Spain) | *Torulaspora delbrueckii* |
| H.S.2 | Whole meal rye  (Albacete, Spain) | *Pichia fermentans* |

433 yeasts isolated from the indicated cereal matrices and the corresponding species identified by molecular genetic techniques (Materials and Methods, 2.2.2, 2.2.3. and 2.2.4.). (1) Type I Mother Doughs (MDs) numbered MD1-1MD14 (Table 1) made by the same baker (E. Mateos, Entrala, Zamora, Spain) and 7 firm MDs numbered MD15-MD21 (Table 1) obtained from other sources. (a) MD15, MD16 and MD17, homemade by M.J. Asensio (MJA, Valladolid, Spain); (b) MD18 and MD19, made by J.A. Ribas (Forn Cruixent, Barcelona, Spain); and (c) MD20 and MD21, from travelling baker and Fred Bakeries (Boulogne-sur-Mer, France). The MD consistencies and back slopping steps BS1 (~one week) or FP s (~one month) are given when known (Materials and Methods, 2.1.1). The whole meal flours are indicated as WM and all the others were refined flours. (2) Yeast isolates from 16 bakery doughs BD1-1BD16, made from wheat flours and obtained from towns and provinces in the autonomous region of Castilla y León, Spain (Table 2). **(3)** Yeast isolates from 10 types of grains and 20 flours from different sources (Table 3). A Laboratory Code was assigned to each yeast isolate and were stored as the **PANLEV Yeast Collection** at the Institute of Functional Biology and Genomics (IBFG), CSIC-University of Salamanca, (Spain) and the Department of Microbiology and Genetics University of Salamanca, (Spain).

**Table S2.** Results of the identification of yeast species in the 433 isolates from this study

| **Yeast strains**  **by laboratory code** | **ITS type** | **RAPD group** | **Closest type strain** | **ITS**  **sim. (%)** | **D1/D2**  **sim.**  **(%)** |
| --- | --- | --- | --- | --- | --- |
| P7FP2, P7FP3, **P7FP8**, P7FP9, P7FP10 | I | A | *Kazachstania bulderi* CBS 8638^T^ | 100 | 100 |
| **P6FP8** | I | B | *Kazachstania bulderi* CBS 8638^T^ | 100 | 100 |
| P3FP10, P4FP3, **P5FP3**, P5FP5, P5FP7, P6FP9 | I | C | *Kazachstania bulderi* CBS 8638^T^ | 100 | 100 |
| **P4FP5** | I | D | *Kazachstania bulderi* CBS 8638^T^ | 100 | 100 |
| P5FP4, P5FP6, **P5FP8** | I | E | *Kazachstania bulderi* CBS 8638^T^ | 100 | 100 |
| **P5FP10** | I | F | *Kazachstania bulderi* CBS 8638^T^ | 100 | 100 |
| **P6FP2**, P6FP7, P7FP1 | I | G | *Kazachstania bulderi* CBS 8638^T^ | 100 | 100 |
| **MTB-1**, MTB-2, MTB-3, MBE-1, MBE-2; MBE-3, MBE-4 | I | H | *Kazachstania humilis* CBS 5658^T^ | 97.7 | 100 |
| P1FP7, **P1FP9**, P1FP10, P2FP2 | I | I | *Kazachstania bulderi* CBS 8638^T^ | 100 | 100 |
| P1FP6, P1FP8, P2FP5, P2FP9, **P3FP5**, P3FP6, P3FP8, P4FP6, P4FP8, P5FP1, P5FP2 | I | J | *Kazachstania bulderi* CBS 8638^T^ | 100 | 100 |
| **P1FP2** | I | K | *Kazachstania bulderi* CBS 8638^T^ | 100 | 100 |
| ME2A5, ME3A5, ME3A6, ME4A4, ME5A3, ME5A6, ME6A3, ME7A8, **H4.1** | I | L | *Kazachstania servazzii* CBS 4311^T^ | 99.9 | 99.8 |
| P1J10, P2A1, P2A2, P2A3, P2A4, **P2A5**, P2A6, P2A7, P2A8, P2A9, P2A10, P4A1, P4A4 | I | M | *Kazachstania servazzii* CBS 4311^T^ | 99.6 | 99.8 |
| P4A2, P4A3, P4A5, **P4A6**, P4A7, P4A8, P4A9, P4A10 | II | A | *Meyerozyma guilliermondii* CBS 2030^T^ | 100 | 99.8 |
| H6.1, **H6.3** | II | B | *Meyerozyma carpophila* CBS 5256^T^ | 99.8 | 99.8 |
| **GTi7**, GTi9 | II | C | *Meyerozyma guilliermondii* CBS 2030^T^ | 100 | 99.8 |
| **Ag2**, YMAS59 | II | D | *Meyerozyma carpophila* CBS 5256^T^ | 100 | 99.8 |
| **ME2FP5**, ME2FP6, ME2FP9 | III | A | *Pichia fermentans* CBS 187^T^ | 98.4 | 99.5 |
| **H5.2** | III | B | *Pichia fermentans* CBS 187^T^ | 100 | 99.5 |
| H5.1, ME3A4, P6FP1, P6FP3, **P6FP4**, P6FP5, P6FP6, P7FP4, P7FP5, P7FP6, P7FP7 | III | C | *Pichia fermentans* CBS 187^T^ | 100 | 99.5 |
| **ME2A1**, ME2A4, ME4A1, ME6A8, P6FP10 | III | D | *Pichia fermentans* CBS 187^T^ | 100 | 99.5 |
| ME4FP10, **ME4A7** | III | E | *Pichia fermentans* CBS 187^T^ | 100 | 99.5 |
| H2.1, ME4A5, **ME2FP2** | III | F | *Pichia fermentans* CBS 187^T^ | 99.8 | 99.5 |
| ME1A2, **ME1A5**, ME1A7, ME4A3 | III | G | *Pichia fermentans* CBS 187^T^ | 99.8 | 99.5 |
| **HRTi8** | III | H | *Pichia fermentans* CBS 187^T^ | 98.4 | 99.5 |
| **ME3A1** | III | I | *Pichia fermentans* CBS 187^T^ | 99.8 | 99.5 |
| **HRTi7**, ME5A1, YMATi2, YMATi3, YMATi4 | III | J | *Pichia fermentans* CBS 187^T^ | 98.4 | 99.5 |
| P1J4, P1J9, P1FP3, P1FP5, P2FP1, P2FP3, P2FP4, P2FP6, P2FP7, P2FP8, **P3FP2**, P3FP3, P3FP4, P3FP7, P3FP9, P4FP1, P4FP2, P4FP4, P4FP7, P4FP9, P4FP10, P5FP9 | III | K | *Pichia fermentans* CBS 187^T^ | 100 | 99.5 |
| **EE9B**, H.S.2 | III | L | *Pichia fermentans* CBS 187^T^ | 99.1 | 99.5 |
| **EE1A**, EE5A | III | M | *Pichia fermentans* CBS 187^T^ | 100 | 99.5 |
| SFG1, SFG2, SFG3, SFG9, SFG10, MFb1, MFb2, MFb3, MFb4, MFb5, MFb6, MFb7, MFb8, MFa1, MFa2, MFa3, MFa4, MFa5, MFa6, MFa7, Bc4, Bc5, Bc6, Bc7, Ent1, Ent3, Ent4, Ent5, Ent6, Ent7, Vid1, Vid2, Vid3, Vid4, Vid5, Br1, Br2, Br3, Br4, Br5, Br6, Br7, Br8, Br9, Br10, Ay1, Ay2, Ay3, Ay4, Ay5, Ay6, Ag1, Ag3, Ag4, Ag5, Ag6, Ag7, Gal1, Gal2, Gal3, Gal4, Gal5, Gal6, ME1FP2, ME3FP2, ME3FP10, ME4FP2, ME7FP1, ME7FP2, ME7FP3, ME7FP4, LEV53, LEV54, P1J2, P1J3, P1J5, P1J6, P1J7, P1J8, P3D1, P3D10, P1FP1, P1FP4, YMAS4, YMAS5, YMAS6, YMAS7, YMAS8, YMAS9, YMAS10, YMAS11, **YMAS12**, YMAS13, YMAS14, YMAS15, YMAS16, YMAS18, YMAS19, YMAS20, YMAS21, YMAS22, YMAS23, YMAS24, YMAS27, YMAS28, YMAS29, YMAS31, YMAS32, YMAS34, YMAS35, YMAS36, YMAS38, YMAS39, YMAS41, YMAS42, YMAS43, YMAS44, YMAS45, YMAS46, YMAS47, YMAS48, YMAS49, YMAS52, YMAS55, YMAS57, YMAS60, YMAS61, YMAS62, YMAS63 | IV | A | *Saccharomyces cerevisiae* CBS 1171^T^ | 100 | 100 |
| **ME1A8**, ME3A8, ME6A2, ME7A2 | IV | B | *Saccharomyces cerevisiae* CBS 1171^T^ | 100 | 100 |
| Ent2, LEV55, LEV56, LEV57, LEV58, ME5FP9, P7F1, P7F2, P7F4, P3FP1, **YMAS3** | IV | C | *Saccharomyces cerevisiae* CBS 1171^T^ | 99.9 | 100 |
| YMATi1, YMATi5, **GTi5** | IV | D | *Saccharomyces cerevisiae* CBS 1171^T^ | 99.6 | 100 |
| **MJA2.1**, MJA2.2 | IV | E | *Saccharomyces cerevisiae* CBS 1171^T^ | 99.8 | 100 |
| YMAS1, **YMAS2** | IV | F | *Saccharomyces cerevisiae* CBS 1171^T^ | 99.9 | 100 |
| ME7FP5, ME7FP6, ME7FP7, ME7FP8, ME7FP9, ME7FP10, **ME5FP10** | IV | G | *Saccharomyces cerevisiae* CBS 1171^T^ | 99.8 | 100 |
| **ME3FP9**, ME6FP5 | V | A | *Torulaspora delbrueckii* CBS 1146^T^ | 100 | 100 |
| ME2A7, ME2A8, ME5A4, **ME5A5** | V | B | *Torulaspora delbrueckii* CBS 1146^T^ | 100 | 100 |
| P3D5, **P3D6**, P7F9 | V | C | *Torulaspora delbrueckii* CBS 1146^T^ | 100 | 100 |
| P6A4, P6A6, P6A7, P6A8, P6A9, **P6A10** | V | D | *Torulaspora delbrueckii* CBS 1146^T^ | 100 | 100 |
| P6A3, P6A5, **P7F10** | V | E | *Torulaspora delbrueckii* CBS 1146^T^ | 100 | 100 |
| P3D2, P3D4, P3D7, P3D8, P5A3, P5A4, P5A5, P5A6, **P5A7**, P5A10, P6A2, P7F3, P7F5, P7F6, P7F7, P7F8 | V | F | *Torulaspora delbrueckii* CBS 1146^T^ | 100 | 100 |
| **P5A8** | V | G | *Torulaspora delbrueckii* CBS 1146^T^ | 100 | 100 |
| **P5A2**, P5A9, P6A1, P1J1, P3D3, P3D9 | V | H | *Torulaspora delbrueckii* CBS 1146^T^ | 100 | 100 |
| H3.3, ME5A7, ME3FP4, ME4FP5, ME4FP7, ME4FP8, ME5FP4, ME5FP7, ME6FP1, ME6FP2, ME6FP3, ME6FP4, ME6FP6, ME6FP7, ME6FP8, ME6FP9, **ME6FP10** | V | I | *Torulaspora delbrueckii* CBS 1146^T^ | 100 | 100 |
| H1.2, ME1FP6, ME1FP7, **ME2FP10**, ME4FP3, ME4FP6 | V | J | *Torulaspora delbrueckii* CBS 1146^T^ | 99.9 | 100 |
| ME3FP1, **ME3FP3** | V | K | *Torulaspora delbrueckii* CBS 1146^T^ | 99.9 | 100 |
| H2.2, H3.2, ME2A6, **ME3A7**, ME4A2, ME5A2, ME7A5, ME5A8, ME7A1 | V | L | *Torulaspora delbrueckii* CBS 1146^T^ | 100 | 100 |
| **ME3A2**, ME3A3, P5A1 | V | M | *Torulaspora delbrueckii* CBS 1146^T^ | 100 | 100 |
| ME4A6, ME7A3, ME7A4, ME7A6, ME7A7, ME1FP1, ME1FP3, ME1FP4, ME1FP5, ME1FP8, ME2FP4, **ME2FP7** | V | N | *Torulaspora delbrueckii* CBS 1146^T^ | 99.9 | 100 |
| **H.S.1.1**, ME1A1, ME1A3, ME1A6, ME2A3, ME6A1, ME6A4, ME3FP5, ME3FP6, ME3FP7, ME3FP8, ME4FP4, ME4FP9, ME5FP1, ME5FP2, ME5FP3, ME5FP5 | V | O | *Torulaspora delbrueckii* CBS 1146^T^ | 100 | 100 |
| **ME4FP1** | V | P | *Torulaspora delbrueckii* CBS 1146^T^ | 99.9 | 100 |
| **ME2A2**, ME6A5, ME6A6, ME6A7 | V | Q | *Torulaspora delbrueckii* CBS 1146^T^ | 100 | 100 |
| ME1A4, **ME5FP6** | V | R | *Torulaspora delbrueckii* CBS 1146^T^ | 99.9 | 100 |
| EE6A, EE6C, **T9**, T11, T12, T13 | VI | A | *Wickerhamomyces anomalus* CBS 5759^T^ | 100 | 100 |
| **ME5FP8** | VI | B | *Wickerhamomyces anomalus* CBS 5759^T^ | 98.7 | 100 |
| **GTi1**, GTi4, HRTi1, HRTi3, HRTi6, YMAT1, YMAT2 | VI | C | *Wickerhamomyces anomalus* CBS 5759^T^ | 100 | 100 |
| **ME2FP3**, ME2FP8 | VI | D | *Wickerhamomyces anomalus* CBS 5759^T^ | 100 | 100 |
| **ME1FP9**, ME1FP10, ME2FP1 | VI | E | *Wickerhamomyces anomalus* CBS 5759^T^ | 100 | 100 |

In bold, the yeast isolates selected from each RAPD group for identification.

**Table S3.** Species and number of yeasts isolated from MDs relative to the flour, dough consistency and fermentation time.

| **(a)**  **Fermentation time** | | |  |  | | | **7 Firm MDs** | | | | | | | | |  |  | **7 Liquid MDs** | | | | | | | | | |  | | |  |  |  |  |
| --- | --- | --- | --- | --- | --- | --- | --- | --- | --- | --- | --- | --- | --- | --- | --- | --- | --- | --- | --- | --- | --- | --- | --- | --- | --- | --- | --- | --- | --- | --- | --- | --- | --- | --- |
|  |  |  |  | **MD1**  W180-200 | **MD3**  W130-150 | | | **MD5**  TZM | **MD7**  WMW | | **MD9**  Tr | **MD11**  WMTr | **MD13**  Oromas | | |  |  | **MD2**  W180-200 | | **MD4**  W130-150 | **MD6**  TZM | | | **MD8**  WMW | **MD10**  Tr | | **MD12**  WMTr | | **MD14**  MO6F |  | |  |  |  |
| **BS1** (~one week)) | | |  | *Pf* 3 | *Pf* 2 | | | *Pf* 1 | *Pf* 2 | | -- | *Pf* 1 | *Pf* 4 | | |  |  | *Pf* 2 | | *--* | *--* | | | *--* | *--* | | *--* | | *--* |  | |  |  |  |
|  |  |  |  | *Td* 4 | *Td* 3 | | | *Td* 5 | *Td 5* | | *Td* 6 | *Td* 5 | *Td* 2 | | |  |  | *Td* 1 | | *Td* 8 | *--* | | | *--* | *Td* 10 | | *Td* 10 | | *Td* 7 |  | |  |  |  |
|  |  |  |  | -- | *Ks* 2 | | | *Ks* 2 | *Ks* 1 | | *Ks* 1 | *Ks* 1 | *Ks* 1 | | |  |  | *Ks* 1 | | *--* | *Ks* 2 | | | *Ks* 10 | -- | | -- | | -- |  | |  |  |  |
|  |  |  |  | *S*c 1 | *Sc* 1 | | | -- | -- | | *Sc* 1 | *Sc* 1 | -- | | |  |  | *Sc* 6 | | *Sc* 2 | -- | | | -- | -- | | -- | | *Sc 3* |  | |  |  |  |
|  | | |  | -- | *--* | | | -- | -- | | *--* | -- | -- | | |  |  | *--* | | -- | *Mg* 8 | | | *--* | -- | | -- | | -- |  | |  |  |  |
| **FP** (~one month) | | |  | *--* | *--* | | | *--* | *P f4* | | *--* | *--* | *Pf* 1 | | |  |  | *Pf* 2 | | *Pf* 5 | *Pf* 6 | | | *Pf* 6 | *Pf* 6 | | *Pf* 1 | | *Pf* 4 |  | |  |  |  |
|  |  |  |  | *Td* 7 | *Td* 8 | | | *Td* 7 | *Td* 3 | | *--* | *Td* 10 | *Td* 8 | | |  |  | *--* | | *--* | *--* | | | *--* | *--* | | *--* | | *--* |  | |  |  |  |
|  |  |  |  | *Wa* 2 | *--* | | | *Wa* 1 | *Wa* 3 | | *--* | *--* | *--* | | |  |  | *Kb 6* | | *Kb* 4 | *Kb* 4 | | | *Kb* 3 | *Kb* 4 | | *Kb* 9 | | *Kb* 6 |  | |  | |  |
|  |  |  |  | *Sc* 1 | *Sc* 2 | | | *Sc* 2 | *--* | | *Sc* 10 | *--* | *Sc* 1 | | |  |  | *Sc* 2 | | *Sc* 1 | -- | | | -- | -- | | -- | | -- |  | |  |  |  |
| **(b)** | |  | **7 Firm MD_S_** | | | | | | | | | | | | | | | | | | | | | | | | | | | | | | |  |
|  | |  | **MD15** | | | | **MD16** | | | | **MD17** | | | |  | **MD18** | | | | **MD19** | | |  | **MD20** | | | **MD21** | | | | | | |  |
| **Fermentation time** | |  | W | | | | Tr | | | | WMTr | | | |  | Tr | | | | WMW | | |  | W | | | W | | | | | | |  |
| **BS1** (~one week) | |  |  | | | | *Wa* 2 | | | | *Pf* 3 | | | |  |  | | | |  | | |  |  | | |  | | | | | | |  |
| **FP** (~one month) | |  | *Sc* 2 | | | |  | | | | *Sc* 2 | | | |  | *Sc* 4 | | | | | | |  |  | | |  | | | | | | |  |
|  |  |  |  | | | |  | | | |  | | | |  | *Kh* 3 | | | | *Kh* 4 | | |  | *Sc* 7 | | | *Sc* 8 | | | | | | |  |

14 Type I MDs made by baker E. M, (Zamora, Spain) and **(b)** 7 firm MDs from other origins, all made of wheat flours (Table 1). W180–200, W130–150, TZM and Oromas, wheat flours; tr and WMtr, refined and whole meal tritordeum, respectively; WMW, whole meal wheat; MO6F mix of 6 flours. Yeast species and isolate numbers are indicated, and the species abbreviated as follows: *Pichia fermentans, Pf;* *Torulaspora delbrueckii, Td*; *Kazachstania. servazzii, Ks*; *Saccharomyces cerevisiae*; *Sc;* *Meyerozyma guilliermondii, Mg*; *Wickerhamomyces anomalus, Wa*; *Kazachstania bulderi, Kb*; *Kazachstania humilis, Kh*. Colors used to highlight the species isolated from specific MDs: dark grey = firm and liquid at BS1; yellow = liquid at BS1; light grey = liquid (MD6) at BS1; salmon = firm at FP; and brown = liquid at FP.

**Table S4.**  The 81 discarded isolates and matrices of origin

| **Sample** | | | **Source** | **Discarded (isolates)** | | | **Species** |
| --- | --- | --- | --- | --- | --- | --- | --- |
| **Grain** | | |  |  | | |  |
| EE9 | | oat (Emilio Esteban, Valladolid, Spain) | | | 1 (2) | ND | |
| T1 | | tritordeum (Exp. crop, Salamanca, Spain) | | | 2 (2) | ND | |
| T7 | | Tritordeum-bulel (Córdoba, Spain) | | | 1 (1) | ND | |
| GTit | | tritordeum (Italy) | | | 2 (9) | *Aureobasidium pullulans* | |
| GTit | | tritordeum (Italy) | | | 1 (9) | *Rhodotorula glutinosa* | |
| GTit | | tritordeum (Italy) | | | 1 (9) | *ND* | |
| **Flour** | |  | | |  |  | |
| H1 | | wheat (W180-200) (Zamora, Spain) | | | 1 (2) | *Cryptococcus albidus* | |
| H3 | | wheat (W130-150) (Zamora, Spain) | | | 1 (3) | *Cryptococcus albidus* | |
| H6 | | Whole meal tritordeum (WMtr) (Zamora, Spain) | | | 1 (3) | ND | |
| Ytrum | | | tritordeum –aucan | | 31 (31) | ND | |
| T9 | | tritordeum –aucan | | | 1 (5) | ND | |
| ACoFa | | tritordeum–aucan (Córdoba, Spain) | | | 3 (3) | ND | |
| AJFa | | tritordeum–aucan (Jerez, Spain) | | | 1 (1) | ND | |
| BCFb | | tritordeum–bulel (Sevilla, Spain) | | | 2 (4) | *Candida parapsilopsis* | |
| BCFb | | tritordeum–bulel (Sevilla, Spain) | | | 2 (4) | ND | |
| BCoFa | | tritordeum–bulel (Córdoba, Spain) | | | 5 (5) | ND | |
| BJFb | | tritordeum–bulel (Jerez, Spain) | | | 2 (2) | ND | |
| HRTit | | tritordeum (Italy) | | | 1 (6) | ND | |
| THI | | Whole meal tritordeum | | | 5 (5) | ND | |
| ACoI | | Whole meal tritordeum-aucan (Córdoba, Spain) | | | 2 (2) | ND | |
| BCI | | tritordeum–bulel (Sevilla, Spain) | | | 1 (1) | ND | |
| BCoI | | Whole meal tritordeum–bulel (Córdoba, Spain) | | | 2 (2) | ND | |
| HRITit | | Whole meal tritordeum (Italy) | | | 2 (2) | ND | |
| **Fermented matrix** |  | | | |  |  | |
| SFG | BD3 (Salamanca, Spain) | | | | 9 (14) | ND | |
| MMB | MD19 (Barcelona, Spain) | | | | 1 (1) | ND | |

ND: non identified species (development of hyphae)
